# Molecular mimicking of C-terminal phosphorylation tunes the surface dynamics of Ca_V_1.2 calcium channels in hippocampal neurons

**DOI:** 10.1074/jbc.M117.799585

**Published:** 2017-11-27

**Authors:** Alessandra Folci, Angela Steinberger, Boram Lee, Ruslan Stanika, Susanne Scheruebel, Marta Campiglio, Claudia Ramprecht, Brigitte Pelzmann, Johannes W. Hell, Gerald J. Obermair, Martin Heine, Valentina Di Biase

**Affiliations:** From the ‡ Institute of Biophysics, Medical University of Graz, 8010 Graz, Austria,; the § Department of Pharmacology, University of California, Davis, California 95616,; the ¶ Department of Physiology and Medical Physics, Medical University of Innsbruck, 6020 Innsbruck, Austria, and; the ‖ Leibniz Institute for Neurobiology, 39118 Magdeburg, Germany

**Keywords:** dihydropyridine receptor (DHPR), fluorescence, imaging, molecular dynamics, neuron, trafficking, L-type Ca^2+^ channels, Ser1700, Ser1928, excitatory

## Abstract

L-type voltage-gated Ca_V_1.2 calcium channels (Ca_V_1.2) are key regulators of neuronal excitability, synaptic plasticity, and excitation-transcription coupling. Surface-exposed Ca_V_1.2 distributes in clusters along the dendrites of hippocampal neurons. A permanent exchange between stably clustered and laterally diffusive extra-clustered channels maintains steady-state levels of Ca_V_1.2 at dendritic signaling domains. A dynamic equilibrium between anchored and diffusive receptors is a common feature among ion channels and is crucial to modulate signaling transduction. Despite the importance of this fine regulatory system, the molecular mechanisms underlying the surface dynamics of Ca_V_1.2 are completely unexplored. Here, we examined the dynamic states of Ca_V_1.2 depending on phosphorylation on Ser-1700 and Ser-1928 at the channel C terminus. Phosphorylation at these sites is strongly involved in Ca_V_1.2-mediated nuclear factor of activated T cells (NFAT) signaling, long-term potentiation, and responsiveness to adrenergic stimulation. We engineered Ca_V_1.2 constructs mimicking phosphorylation at Ser-1700 and Ser-1928 and analyzed their behavior at the membrane by immunolabeling protocols, fluorescence recovery after photobleaching, and single particle tracking. We found that the phosphomimetic S1928E variant increases the mobility of Ca_V_1.2 without altering the steady-state maintenance of cluster in young neurons and favors channel stabilization later in differentiation. Instead, mimicking phosphorylation at Ser-1700 promoted the diffusive state of Ca_V_1.2 irrespective of the differentiation stage. Together, these results reveal that phosphorylation could contribute to the establishment of channel anchoring mechanisms depending on the neuronal differentiation state. Finally, our findings suggest a novel mechanism by which phosphorylation at the C terminus regulates calcium signaling by tuning the content of Ca_V_1.2 at signaling complexes.

## Introduction

In neurons activation of L-type voltage-gated Ca_V_1.2 calcium channels (Ca_V_1.2) serves excitation-transcription coupling, long-term potentiation, spatial memory, and synaptic plasticity (1–3). Ca_V_1.2 is embedded in the neuronal membrane within macromolecular signaling complexes whose integrity preserves the efficiency of the underlying signaling pathways. However, the molecular mechanisms controlling channel trafficking, targeting, and density at signaling domains are poorly understood.

Membrane-expressed Ca_V_1.2 channels distribute in clusters on the soma and along the dendrites of cultured hippocampal neurons (4). Here, individual channels are variably subject to lateral diffusion within the lipid bilayer. Clustered channels exhibit highly restricted spatial mobility consistent with their anchoring to the membrane microdomains below (5). These stably clustered channels dynamically exchange with a population of non-clustered diffusive ones to preserve a local steady-state (5). Thus, regulation of Ca_V_1.2 lateral mobility is an uncharted mechanism to potentially adjust the channel content at cluster sites.

Ca_V_1.2 has a long intracellular C-tail accounting for about one-third of the whole channel sequence and acting as a molecular hub to signaling molecules and scaffold proteins. The two C-terminal Ser-1700 and Ser-1928 phosphorylation sites were shown to be critical for adrenergic regulation of Ca_V_1.2-mediated calcium currents, for synaptic plasticity, and for Ca_V_1.2 signaling to the nucleus. Maintenance of phosphorylated Ser-1700 (pSer-1700) and Ser-1928 (pSer-1928) is necessary for efficient activation of NFAT signaling via Ca_V_1.2 in cultured neurons (6, 7). Furthermore, phosphorylation on Ser-1928, but not Ser-1700, is required for a Ca_V_1.2-mediated long-term potentiation induced by a prolonged θ tetanus (8). Finally, pSer-1928 displaces the β_2_ adrenergic receptors from Ca_V_1.2 desensitizing the channel for highly localized adrenergic signaling (9).

Direct phosphorylation of post-synaptic ion channels or their interacting proteins is known to regulate their function, trafficking, and stabilization at synapses. These notions prompted us to examine whether pSer-1700 and pSer-1928 impact the surface trafficking of Ca_V_1.2 in neurons. By using a molecular phosphomimetic approach we found that selective phosphorylation on Ser-1928 and/or Ser-1700 regulates the amount, distribution, and lateral mobility of membrane-inserted Ca_V_1.2 according to the neuronal differentiation state. Thus, by modulating the channel surface level and dynamics, Ca_V_1.2 C-terminal phosphorylation represents a potential mechanism to tune calcium signaling in response to neuromodulation.

## Results

### Reproducing constitutive phosphorylation at Ser-1700 and Ser-1928 alters the number and size of Ca_V_1.2 clusters in cultured hippocampal neurons

**Figure 1.**
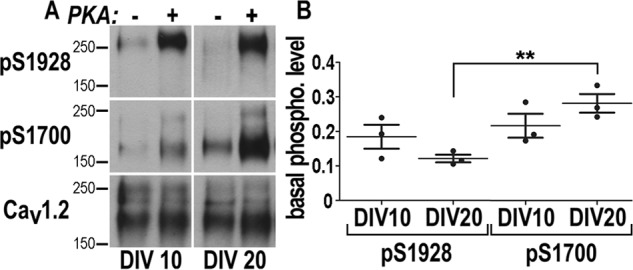
**Quantification of Ser-1700 and Ser-1928 phosphorylation in cultured hippocampal neurons.** Hippocampal neurons were extracted at 10 and 20 DIV, each sample was split into equal pairs, and Ca_V_1.2 was immunoprecipitated. One sample of each pair but not the other was phosphorylated *in vitro* with purified PKA catalytic subunit under conditions that lead to near complete phosphorylation of the PKA site on Ca_V_1.2, as quantified earlier (36). *A,* samples of sequential probing of immunoblots with antibodies against pSer-1928, pSer-1700, and finally total Ca_V_1.2. *B,* immunosignals were quantified by film densitometry. pSer-1700 and pSer-1928 signals were corrected for differences in relative amounts of Ca_v_1.2. Scatter plots show pSer-1700 and pSer-1928 levels detected in untreated samples as a fraction of the paired maximally phosphorylated samples. Of the Ser-1928 sites 18.4 ± 4.9% were phosphorylated in hippocampal cultures at 10 DIV and 12.1 ± 1.6% at 20 DIV. Of the Ser-1700 sites 21.6 ± 4.9% were phosphorylated at 10 DIV and 28.1 ± 3.8% at 20 DIV. All errors are given as S.D. Statistics: ANOVA: **, *p* = 0.03; *n* = 3 obtained in three independent experiments.

**Figure 2.**
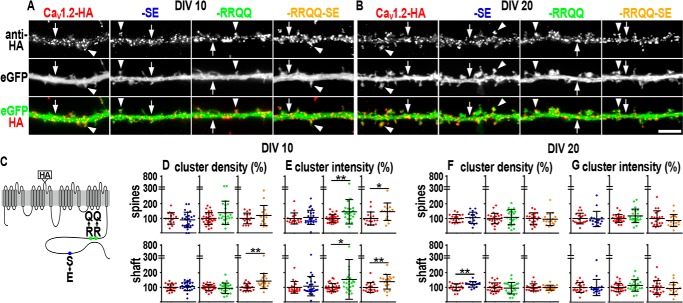
**Distribution and size of clusters of membrane-expressed phosphomimetic Ca_V_1.2-HA constructs.** *A* and *B,* dendritic segments of neurons transfected with Ca_V_1.2-HA, Ca_V_1.2-HA-SE, Ca_V_1.2-HA-RRQQ, or Ca_V_1.2-HA-RRQQ-SE and immunolabeled with anti-HA antibody at DIV 10 (*A*) and DIV 20 (*B*) in non-permeabilized conditions. Co-expressed soluble eGFP outlines the morphology of the dendrites. All channel mutants distribute in clusters along the dendritic shafts (*arrows*) and on the spines (*arrowheads*) similar to the control Ca_V_1.2-HA. *Scale bar*, 5 μm. *C,* topology of Ca_V_1.2-HA indicating the position of the extracellular HA tag, the S1928E exchange (SE, *blue circle*), and the double substitution R1696Q,R1697Q (RRQQ, *green circle*), which prevents the inhibitory interaction within the C terminus (15). *Yellow* represents the Ca_V_1.2-HA carrying the RRQQ and SE mutations (Ca_V_1.2-HA-RRQQ-SE). *D* and *F*, quantification of density (number of clusters per spine or per surface unit of dendritic shaft) and the intensity (average gray values). *E* and *G,* Ca_V_1.2-HA-SE, Ca_V_1.2-HA-RRQQ, and Ca_V_1.2-HA-RRQQ-SE expressed as percentage of control Ca_V_1.2-HA. Cluster density increased along the shaft at DIV 10 for Ca_V_1.2-HA-RRQQ-SE (*D*, *yellow scatter plot*, *p* = 0.002) and at DIV20 for Ca_V_1.2-HA-SE (*F*, *blue scatter plot*, *p* = 0.008). Cluster fluorescence intensity augmented on the spines and along the dendritic shafts of young neurons expressing Ca_V_1.2-HA-RRQQ (*E*, *green scatter plot*, *p* = 0.004 and *p* = 0.017, respectively) and Ca_V_1.2-HA-RRQQ-SE (*E*, *yellow scatter plot*, *p* = 0.018 and *p* = 0.009, respectively). Statistics: Mann-Whitney rank sum test. Analyzed dendritic segments in at least three separate experiments: DIV 10, N_CaV1.2-HA_ = 18–19 and N_CaV1.2-HA-SE_ = 24–26, N_CaV1.2-HA_ = 30 and N_CaV1.2-HA-RRQQ_ = 23, N_CaV1.2-HA_ = 18–19 and N_CaV1.2-HA-RRQQ-SE_ = 15–18; DIV 20, N_CaV1.2-HA_ = 19 and N_CaV1.2-HA-SE_ = 17, N_CaV1.2-HA_ = 26 and N_CaV1.2-HA-RRQQ_ = 23, N_CaV1.2-HA_ = 21–22 and N_CaV1.2-HA-RRQQ-SE_ = 18–20. Scatter plots: mean ± S.D. **, 0.002 < *p* < 0.009 and *, 0.017 < *p* < 0.018.

To determine basal phosphorylation levels of Ser-1700 and Ser-1928, Ca_V_1.2 channels were extracted from young and mature hippocampal cultures at 10 and 20 DIV,^5^ respectively (10, 11), in the presence of potent phosphatase inhibitors to prevent dephosphorylation. Each sample was divided into two equivalent halves and Ca_V_1.2 channels were immunoprecipitated. One half was immediately processed for gel electrophoresis and the other was stoichiometrically phosphorylated with purified PKA before immunoblotting (Fig. 1*A*). Quantification of immunosignals indicated that the phosphorylation level of Ser-1928 was about 18 and 12% and for Ser-1700 22 and 28% at 10 and 20 DIV, respectively. The different values between Ser-1700 and Ser-1928 at DIV 20 suggest that these residues could be subject to distinct regulation and independently targeted by phosphorylation (Fig. 1*B*, DIV 20; ANOVA, *p* = 0.03). To assess whether pSer-1928 and pSer-1700 regulate the distribution of Ca_V_1.2 at the dendritic membrane we generated Ca_V_1.2 mutants mimicking pSer-1700 or pSer-1928, or both, and analyzed their surface expression by immunolabeling experiments in young and mature cultured hippocampal neurons, at DIV 10 and 20, respectively (10, 11). Phosphomimetic mutations were inserted into a previously used and functionally integer Ca_V_1.2 construct carrying an HA tag in the extracellular loop after the transmembrane helix IIS5 (Ca_V_1.2-HA, Fig. S1 and Fig. 2*c*) (4, 5, 12–14). The accessibility of the HA epitope from the extracellular space allows observing exclusively membrane-inserted channel constructs in unpermeabilized conditions. Phosphorylation at Ser-1928 was reproduced by introducing a S1928E substitution (Ca_V_1.2-HA-SE, Fig. 2*C*). Phosphorylation at Ser-1700 was previously shown to increase calcium currents by releasing the constitutive inhibitory interaction between the channel proximal and distal C terminus (15). Such an inhibitory complex is established via positively charged Arg^1696–1697^ residues at the proximal C terminus that form salt bridges with aspartic and glutamic acid residues at the distal C terminus (16). Introducing the two neutralizing R1696Q,R1697Q substitutions was shown to release the inhibitory interaction by preventing the formation of those salt bridges responsible for the association of the distal C terminus with the proximal (16). We hypothesized that the release of the inhibitory interaction could modify the conformation of the channel tail, likely affecting intracellular interactions and thus influencing the Ca_V_1.2 channels distribution and stability at their membrane domains. To address this issue we adopted the same strategy of site-directed mutagenesis used by Hulme *et al.* (16) and we produced a Ca_V_1.2-HA carrying the R1696Q,R1697Q double substitution (Ca_V_1.2-HA-RRQQ, Fig. 2*C*). Last, a Ca_V_1.2-HA including all mutations was generated and named Ca_V_1.2-HA-RRQQ-SE. To equalize channel expression all mutants were inserted into the same backbone plasmid optimized for neuronal expression (4, 5, 12–14). Overall the electrophysiological properties of all HA-tagged constructs were similar to those of the untagged channels indicating that the HA tags are unlikely modifying the current properties (Fig. S1). In line with Hulme *et al.* (16), R1696Q,R1697Q containing mutants exhibited larger current density in HEK293 cells consistent with the release of the autoinhibitory interaction within the C terminus induced by the mutations (Fig. S1) (16). Similarly, R1696Q,R1697Q showed a ∼15% larger current in voltage clamp experiments on cultured hippocampal neurons at DIV 10 and 20 (Fig. S2). In contrast, S1928E augmented current density in neurons but not in HEK293 and R1696Q,R1697Q,S1928E increased it in HEK293 but not in neurons (Fig. S2). These differences are addressed under “Discussion.”

To analyze the surface expression of channel mutants, living neurons co-transfected with Ca_V_1.2-HA, Ca_V_1.2-HA-SE, Ca_V_1.2-HA-RRQQ, or Ca_V_1.2-HA-RRQQ-SE, and soluble eGFP were exposed to primary anti-HA antibodies for 30 min, fixed with paraformaldehyde for 5 min, and probed with a secondary antibody (5, 12–14). To avoid the saturation of the expression capacity, neurons were transfected with the minimum amount of cDNA necessary to achieve detectability. Under similar conditions, membrane expression of transfected Ca_V_1.2-HA was previously shown to be decisively regulated by the interaction with the endogenous β subunit in neurons (13). This implies that the action of the endogenous subunits on transfected channels is essential and crucial to modulate channel levels at the surface. Similar to control CaV1.2-HA, all surface-expressed mutants distributed in discrete clusters on spines (*arrowheads*, Fig. 2, *A* and *B*) and along dendritic shafts (*arrows*, Fig. 2, *A* and *B*), indicating that the molecular interactions underlying cluster organization and subcellular localization were preserved. Quantitative surface expression was assessed by measuring the clusters fluorescent intensity and density along dendritic shafts and on spines as previously described (5, 12). Cluster density and intensity indicate, respectively, the amount of clusters and their channel content (size). Both parameters are linked to the amount of channels at the membrane and their trafficking dynamics. The level of surface-expressed Ca_V_1.2 is maintained by the equilibrium of channel membrane insertion and internalization (5, 17). Furthermore, clustered Ca_V_1.2 channels were shown to dynamically exchange with a population of diffusive non-clustered channels by lateral mobility (5). This mobile pool potentially allows short-term adaptation of the cluster size by entering or leaving the clustered state, thereby increasing and reducing the cluster size accordingly (5). Therefore, overall changes of cluster density and intensity imply a modulation of the underlying channel trafficking dynamics.

At first we examined the membrane expression of Ca_V_1.2-HA-SE. At DIV 10 cluster density and intensity were similar to control Ca_V_1.2-HA (Fig. 2, *D* and *E*, scatter plots, *blue circles*). This result could be interpreted in two ways: the channel trafficking dynamics was unaffected by the S1928E or entirely accelerated, or delayed, but concomitantly preserving channel steady-state levels at the membrane similar to controls. At DIV 20 Ca_V_1.2-HA-SE cluster density increased by ∼20% along the dendritic shaft (Fig. 2*F*, scatter plots, *blue circles*, *p* = 0.008) revealing a higher amount of channels at the membrane. It is therefore plausible that S1928E delayed channel internalization or promoted membrane insertion. If S1928E also favored the recruitment of individual channels to cluster sites or their removal, then cluster intensity was expected to increase or diminish, accordingly. We found that cluster intensity was unaltered (Fig. 2, *E* and *G*, *blue circles*) indicating a negligible role of S1928E for such mechanisms in mature neurons.

Next, we analyzed the Ca_V_1.2-HA-RRQQ mutant. At DIV 10 cluster density was comparable to controls (Fig. 2*D*, scatter plots, *green circles*) and intensity increased along the dendritic shafts and on the spines by 55% (*p* = 0.017) and 46% (*p* = 0.004), respectively (Fig. 2, *E*, scatter plots, *green circles*), indicating that the R1696Q,R1697Q mutations did not alter the number of clusters but increased their channel content. Thus, the overall membrane levels of Ca_V_1.2-HA-RRQQ augmented suggesting that channel delivery to the membrane and its internalization may be promoted or delayed, respectively.

Finally, the double mutant Ca_V_1.2-HA-RRQQ-SE showed a ∼41% increased cluster density along the shaft (Fig. 2, *D*, *yellow circles*, *p* = 0.002) and enhanced fluorescence intensity (Fig. 2*E*, shaft 38%, *p* = 0.009; spine 46%, *p* = 0.02) at DIV 10 indicating that new clusters were formed and their channel content increased. These results showed that the surface expression of the double mutant was also increased. No change was observed at DIV 20 for Ca_V_1.2-HA-RRQQ and Ca_V_1.2-HA-RRQQ-SE (Fig. 2, *F* and *G*). These data could indicate that the channel trafficking was not affected by the mutations, but did not exclude a homogeneous acceleration or delay, which did not alter channel cluster density and intensity, as discussed above for Ca_V_1.2-HA-SE in young neurons.

We also found that Ca_V_1.2-HA-S1700E and Ca_V_1.2-HA-S1700E,S1928E mutants exhibited an increase of channel cluster density and intensity at DIV 10 and 20 similar to the R1696Q,R1697Q and R1696Q,R1697Q,S1928E variants, indicating that the RRQQ constructs serve as valid model for phosphorylation (Fig. S3). Expression of phosphomimetic constructs did not alter spine frequency and thickness of dendritic shafts implying that our analysis was not biased by possible changes of the dendritic morphology (Table S1).

Phosphomimetic mutations enhanced channel membrane expression. Conversely, phospho-resistant variants were expected to exhibit lower membrane levels in comparison with Ca_V_1.2-HA, which could still be endogenously phosphorylated. We found that S1700A and S1700A,S1928A did not alter either parameter at DIV 10, but were associated with a reduction of cluster density and/or intensity in shafts and/or spines of mature neurons (Fig. S4, *A, C, G,* and *I*). Ca_V_1.2-HA-S1928A showed a slight reduction of cluster fluorescence intensity on the spines at DIV 20 (Fig. S4*H*). Lack of significant effects in some conditions (Fig. S4, *A–C* and *H*) has to be taken with caution because endogenous phosphorylation could be locally too low to produce an effect above the sensitivity of our acquisition settings. In line with this reasoning, S1700A and S1700A,S1928A constructs produced the significant results at DIV 20 when basal pSer-1700 was stronger (Fig. 1).

Altogether, the results presented above support a potential role for pSer-1700 and pSer-1928 as regulators of membrane expression and distribution of Ca_V_1.2 along the dendrites of hippocampal neurons.

### S1928E and R1696Q,R1697Q substitutions modify the dynamics of dendritic Ca_V_1.2 channels

Surface immunolabeling experiments showed that S1928E and/or R1696Q,R1697Q mutations affected the steady-state levels of Ca_V_1.2 clusters in cultured hippocampal neurons according to the differentiation stage. To address whether the surface expression of channel mutants was dependent on altered channel trafficking dynamics, fluorescence recovery after photobleaching (FRAP) experiments were performed on living neurons expressing N-terminal eGFP-tagged eGFP-Ca_V_1.2, eGFP-Ca_V_1.2-SE, eGFP-Ca_V_1.2-RRQQ, and eGFP-Ca_V_1.2-RRQQ-SE (5, 18). Under these experimental conditions the observed fluorescence included intracellular and membrane-expressed channels. Therefore, FRAP curves represented the channel overall dynamics including intracellular trafficking and lateral mobility within the plane of the membrane (5, 17, 18). Patch clamp recordings in HEK293 showed that eGFP-tagged channels are comparable with the untagged ones indicating that eGFP did not alter the current properties (Fig. S1). Small circular regions of transfected neurons were rapidly bleached with laser pulses and imaged for the following 60 s at 2 Hz. The extent of fluorescence recovery at the onset of FRAP curves estimated the rate at which unbleached channels diffused within the bleached region and described, therefore, the channel dynamic state. The amplitude of the curve represents the fraction of mobile channels concomitantly driven by intracellular and surface traffic.

**Figure 3.**
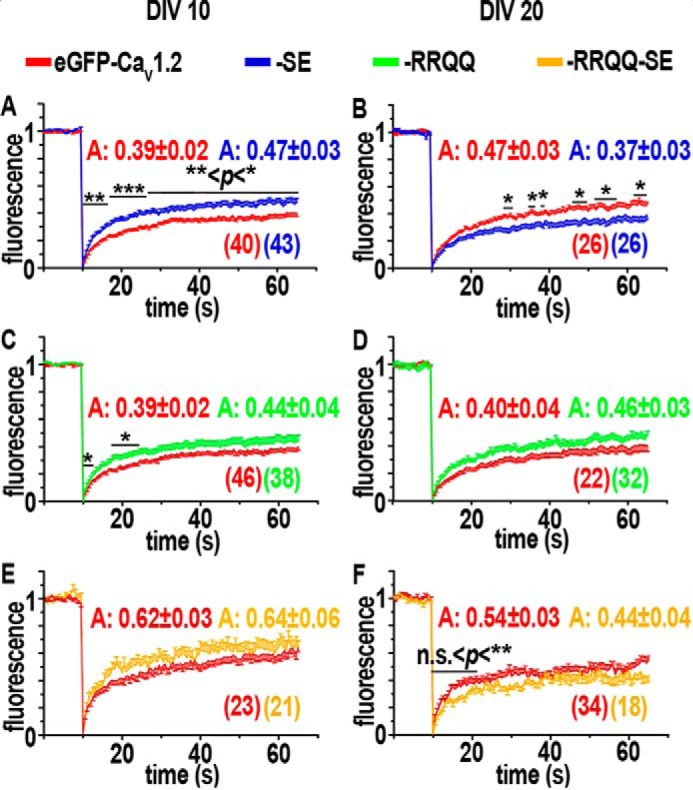
**FRAP analysis in cultured young and mature hippocampal neurons.** Experiments were conducted on neurons overexpressing eGFP-Ca_V_1.2 (*red*), eGFP-Ca_V_1.2-SE (*blue*), eGFP-Ca_V_1.2-RRQQ (*green*), or eGFP-Ca_V_1.2-RRQQ-SE (*yellow*). *A, C,* and *E,* averaged FRAP curves of dendritic regions in young neurons (DIV 10) were higher for all mutants indicating faster dynamics. *B, D*, and *F,* FRAP curves of dendritic regions derived from mature neurons (*DIV* 20). *B,* the lower fluorescence recovery of eGFP-Ca_V_1.2-SE (*blue*) than control eGFP-Ca_V_1.2 (*red*) indicates a slower dynamics and a reduced fraction of mobile channels. Slower dynamics was also observed at the rising phase of eGFP-Ca_V_1.2-RRQQ-SE FRAP curve (*F*). “*A*” indicates the amplitude of curves calculated as mean of the last values of individual curves and then averaged. Two-tailed *t* test: *, 0.1< *p* < 0.5; **, 0.01 < *p* < 0.001; ***, 0.001 < *p* < 0.0001. Data were obtained from three experiments on at least three different cultures. The number of regions analyzed for each construct derived from at least 11 neurons is indicated in *parentheses.* Data are presented as mean ± S.E.

Surface immunolabeling of Ca_V_1.2-HA-SE showed that cluster density and intensity were similar to control Ca_V_1.2-HA (Fig. 2, *D* and *E*, *blue circles*). We verified whether the underlying trafficking mechanisms were unaffected by the S1928E mutation. At DIV 10 the averaged fluorescence values of eGFP-Ca_V_1.2-SE FRAP curves were consistently higher than those of control eGFP-Ca_V_1.2 and the curve amplitude increased from 39 to 47% (Fig. 3*A*, *blue*) revealing that S1928E increased the diffusion rate and fraction of dynamic channels in young neurons. Together with surface immunolabeling experiments (Fig. 2, *D* and *E*, *blue circles*) this result suggests that S1928E accelerated the overall channel trafficking without changing the steady-state level of channels at the membrane. In contrast, at DIV 20 eGFP-Ca_V_1.2-SE FRAP curves exhibited a ∼10% smaller amplitude indicating a reduced extent of the overall trafficking events (Fig. 3*B*, *blue*). Together with the increase of cluster density observed in immunolabeling experiments (Fig. 2*F*, *blue*), this result indicates that S1928E mutants accumulated at the membrane probably upon impairment of channel internalization, similar to previous observations (5). Consistently, we found that, unlike wild-type channels (5), cluster density of Ca_V_1.2-HA-SE was insensitive to blocking the endocytosis (Fig. S5). Finally, the fluorescence values at the rising phase of the eGFP-Ca_V_1.2-SE curve were similar to control eGFP-Ca_V_1.2, indicating that the channel diffusion rate itself was not affected by the mutation in mature neurons (Fig. 3*B*, *blue*).

In surface immunolabeling experiments on young neurons Ca_V_1.2-HA-RRQQ exhibited larger cluster size and higher amounts of channels at the membrane (Fig. 2, *D* and *E*, *green circles*). These data suggest that R1696Q,R1697Q could stabilize channels at cluster sites and that a putative impairment of channel internalization could be responsible for the increased membrane expression. If so, eGFP-Ca_V_1.2-RRQQ FRAP curves were expected to show a lower recovery rate. Surprisingly, the steeper rising phase of the FRAP curve indicated that eGFP-Ca_V_1.2-RRQQ was endowed with a faster diffusion rate (Fig. 3*C*, *green*, 0.1 < *p* < 0.5), which rejects the hypothesis of a stronger stabilizing mechanism. Channels accounting for such a fast recovery are required to be quickly and readily available meaning that lateral mobility of channels at the membrane was likely responsible of this result. Furthermore, we observed that the amplitude of the eGFP-Ca_V_1.2-RRQQ FRAP curve was comparable with control and, therefore, did not show any change of trafficking that could explain the higher amount of channels at the membrane observed in surface immunolabeling experiments (Fig. 2, *D* and *E*). To reconcile our results, we reasoned that enhanced membrane levels of channels could be induced by an impairment of channel endocytosis that would reduce the recovery of fluorescence in FRAP. Thus, a mixed slower intracellular and faster surface dynamics would compete and neutralize each other in FRAP analysis. At DIV 20 eGFP-Ca_V_1.2-RRQQ fluorescence recovery did not show differences with eGFP-Ca_V_1.2 at any time point, meaning that no major change of channel trafficking was detectable in mature neurons under these experimental conditions (Fig. 3*D*, green).

Finally, if the increased channel surface expression induced by R1696Q,R1697Q,S1928E in young neurons (Fig. 2, *D* and *E*, *yellow*) was determined by reinforced channel delivery to the membrane or delayed channel internalization, then the eGFP-Ca_V_1.2-RRQQ-SE FRAP recovery rate was expected to increase or reduce, accordingly. However, the eGFP-Ca_V_1.2-RRQQ-SE was similar to controls at DIV 10 (Fig. 3*E*), which was not supportive of either mechanism. To reconcile these results we reasoned that R1696Q,R1697Q,S1928E could accelerate one traffic process and slow down a second competing one, so that the resulting FRAP curve was unaltered, as discussed for R1696Q,R1697Q in young neurons. At DIV 20 eGFP-Ca_V_1.2-RRQQ-SE showed some modest reduction of fluorescent values at the initial phase of the curve suggesting that the channel dynamics was slightly slower than controls in this condition (Fig. 3*F*, *yellow*). We found that eGFP-Ca_V_1.2-S1700E and eGFP-Ca_V_1.2-S1700E,S1928E mutants exhibited FRAP curves similar to those of the R1696Q,R1697Q and R1696Q,R1697Q,S1928E variants, indicating that the RRQQ constructs serve as valid model for analyzing pSer-1700-dependent channel dynamics (Fig. S3).

The effect of endogenous phosphorylation on channel dynamics was tested using eGFP-tagged phospho-resistant channel constructs compared with control eGFP-Ca_V_1.2 in FRAP experiments. Unexpectedly, all curves were similar to controls (Fig. S4, *D–F* and *J–L*). Because S1700A and S1700A, S1928A mutations were already shown to impact channel trafficking in surface immunolabeling experiments (Fig. S4, *G* and *I*), at least the eGFP-Ca_V_1.2-S1700A and Ca_V_1.2-S1700A, S1928A variants were expected to diverge from controls. Perhaps the levels of endogenous phosphorylation on control eGFP-Ca_V_1.2 were not high enough to determine trafficking effects detectable above the sensitivity of the acquisition settings.

It is worth mentioning that FRAP curves of fluorescently tagged channels are substantially greater in the present study than in Di Biase *et al.* (5). This is to be expected because in our previous study we observed only channels at the membrane, bleached 10-μm segments of dendrites that implies large distances for diffusion, and imaged via optical sections using a confocal microscope. In the present study we observed the fluorescence of intracellular and membrane-expressed channels, bleached about 2–3 μm large circular regions, and used a wide field conventional epifluorescence microscope. These are very different conditions that produce very different recovery rates.

In summary, FRAP analysis on phosphomimetic variants suggests that pSer-1928 could accelerate the overall channel trafficking dynamics in young neurons and delay it in mature ones. In contrast, pSer-1700 could increase channel diffusion in young neurons but not in mature ones. The channel double mutant did not show obvious differences with controls. However, a full interpretation of FRAP experiments would benefit from a comprehensive analysis of surface trafficking events. Indeed, elucidating the lateral mobility of the channels mutants at the membrane would uncover the impact of the intracellular trafficking component embedded in FRAP curves.

### S1928E and R1696Q,R1697Q tune lateral diffusion of individual Ca_V_1.2 channels

**Figure 4.**
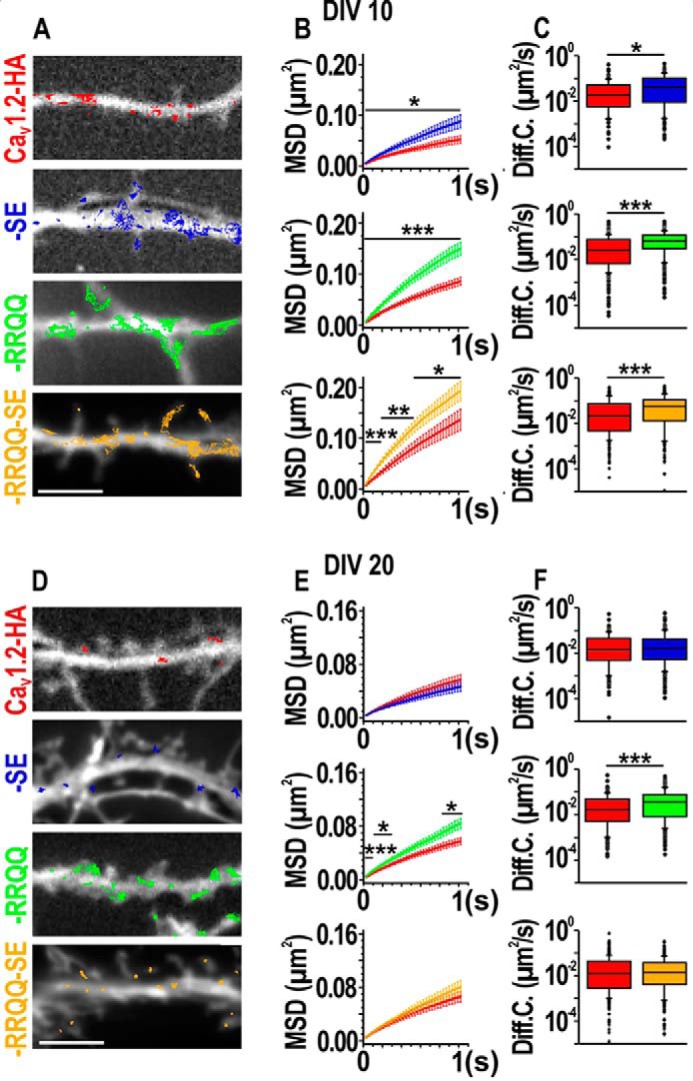
**Single particle tracking analysis of Ca_V_1.2-HA, Ca_V_1.2-HA-SE, Ca_V_1.2-HA-RRQQ, and Ca_V_1.2-HA-RRQQ-SE.** *A* and *D,* representative reconstructed trajectories of QDs bound to channel mutants and superimposed to dendritic segments outlined with soluble eGFP. *Bar*, 5 μm. *B* and *E,* MSD curves of Ca_V_1.2-HA (*red*), Ca_V_1.2-HA-SE (*blue*), Ca_V_1.2-HA-RRQQ (*green*), and Ca_V_1.2-HA-RRQQ-SE (*yellow*) represented as mean ± S.E.; *, 0.01 < *p* < 0.04; **, 0.001 < *p* < 0.009; ***, *p* < 0.001. *C* and *F, box plots* of the diffusion coefficients (median ± interquartile range). Numerical and *n* values are provided in Table 1. Statistics: Shapiro-Wilk normality test failed with *p* < 0.05. Mann-Whitney rank sum test: *, *p* = 0.010; ***, *p* < 0.001.

The differences between FRAP curves at the rising phases suggest that the mobile channels responsible for fluorescence recovery were readily and quickly available. Thus, the lateral diffusion of surface-exposed Ca_V_1.2 was likely responsible for the increased mobility observed in FRAP experiments. To analyze the lateral diffusion of exclusively membrane-expressed Ca_V_1.2 mutants at high spatial and temporal resolution we performed single particle tracking (SPT) analysis (5). Living rat hippocampal neurons transfected with eGFP and HA-tagged Ca_V_1.2 mutants were labeled at low density with rat anti-HA and anti-rat quantum dots (QD) (5). Afterward, QD-labeled neurons were imaged at a frame rate of 30 Hz for 15 s. The position of QDs in each frame was used to reconstruct the trajectories covered by individual particles within the imaging period. Fig. 4, *A* and *D,* shows trajectories of QD-labeled HA-tagged Ca_V_1.2 mutants superimposed to the corresponding eGFP image in young (*A*) and mature neurons (*D*) (19). Visual inspection of trajectories in young neurons suggested that QD-labeled HA-tagged mutants explored larger membrane regions than control Ca_V_1.2-HA (Fig. 4*A*). These differences were shown by plotting the mean squared displacement (MSD) of QDs over time (Fig. 4, *B*). The MSD curves exhibit a negative curvature typical of a confined behavior for all QD-coupled channels and show that the displacement for the mutants was larger than controls (Fig. 4*B*). The diffusion coefficients of individual channels, obtained by linear fitting of the first 4 points of MSD curves, increased for all mutants, indicating higher mobility and a weaker retention at anchoring sites (Fig. 4*C*, Table 1) (19–21). Such faster local dynamics was consistent with the steeper rising phase of eGFP-Ca_V_1.2-SE and eGFP-Ca_V_1.2-RRQQ FRAP curves and supports a potentiation of channel lateral mobility induced by S1928E and R1696Q,R1697Q mutations. The increased diffusion coefficient and lateral mobility of the double mutant was not detected in FRAP curves (Fig. 3*E*, *yellow*) although such a faster dynamics was expected to increase the recovery rat. As proposed above (see previous paragraph), the double mutant likely delayed the channel internalization producing slower intracellular dynamics, which could oppose and neutralize the faster one at the membrane in FRAP analysis. To estimate whether higher diffusion coefficients underlay different modes of channel mobility, trajectories were classified as confined, diffusive, or exchanging between confined and diffusive motions (Fig. S6*A*). For Ca_V_1.2-HA-SE all modes of mobility were essentially similar to controls, whereas for Ca_V_1.2-HA-RRQQ and Ca_V_1.2-HA-RRQQ-SE the diffusive component prevailed. Altogether, these data reveal that the S1928E and R1696Q,R1697Q mutations potentiated Ca_V_1.2 lateral mobility and favored a weaker confinement in young neurons. In mature neurons the trajectories of Ca_V_1.2-HA-SE and Ca_V_1.2-HA-RRQQ-SE were highly confined (Fig. 4*D*, *blue* and *yellow*) similar to controls (*red*) as shown by indistinguishable MSD curves and comparable diffusion coefficients (Fig. 4, *E* and *F,* blue and *yellow*; Table 1). The slightly increased percentage of diffusive trajectories did not affect the overall mobile properties of membrane-expressed Ca_V_1.2-HA-SE channels indicating that S1928E was negligible for regulation of channel lateral mobility in mature neurons (Fig. S6*B*, *blue*). The surface explored by QD-labeled Ca_V_1.2-HA-RRQQ was clearly broader than the control Ca_V_1.2-HA, consistent with the higher displacement described by the MSD functions (Fig. 4, *D* and *E*, *green*). Similar to the R1696Q,R1697Q,S1928E mutant in young neurons, increased lateral mobility of R1696Q,R1697Q and unaltered FRAP curves suggest that this mutation probably slowed down channel internalization in mature neurons. Classification of trajectories showed that for Ca_V_1.2-HA-RRQQ the diffusive motions prevailed (Fig. S6*B*, *green*). As the channel diffusive state corresponds to an extra-clustered state (5), this result should be paralleled by a diminished intensity of Ca_V_1.2-HA-RRQQ cluster intensity consistent with a channel de-clustering mechanism. However, this was not the case (Fig. 2*G*, *green circles*) implying that additional channels were supplied to the membrane to compensate a putative loss of channels from clusters. This is in line with the role proposed above for R1696Q,R1697Q to impair channel internalization.

**Table 1:** **Diffusion coefficients (Diff. Coeff.) of dendritic Ca_V_1.2-HA, Ca_V_1.2-HA-SE, Ca_V_1.2-HA-RRQQ, and Ca_V_1.2-HA-RRQQ-SE**

	DIV 7–9			DIV 15–20		
	*n**^a^*	Diff. Coeff.*^b^* (μm 2 s^−1^)	IQR*^c^*	*n**^a^*	Diff. Coeff.*^b^* (μm 2 s^−1^)	IQR*^c^*
Ca_V_1.2-HA	265	0.0224	0.007/0.059	191	0.0143	0.005/0.045
Ca_V_1.2-HA-SE	217	0.0368*^d^*	0.008/0.092	212	0.0164	0.005/0.042
Ca_V_1.2-HA	457	0.0262	0.007/0.075	174	0.0159	0.005/0.047
Ca_V_1.2-HA-RRQQ	410	0.0638*^e^*	0.030/0.116	259	0.035*^e^*	0.008/0.07
Ca_V_1.2-HA	207	0.0234	0.005/0.083	293	0.0131	0.002/0.044
Ca_V_1.2-HA-RRQQ-SE	223	0.063*^e^*	0.014/0.127	335	0.0141	0.004/0.038

*^a^* Number of trajectories.

*^b^* Median.

*^c^* Interquartile range (0.25–0.75).

*^d^* Mann-Whitney rank sum test, *p* = 0.01.

*^e^* Mann-Whitney rank sum test, *p* < 0.001.

MSD curves and diffusion coefficient in this manuscript are much larger than those observed in our previous study (5). In the present study we analyzed QD-labeled channels all over the dendrites in neurons co-transfected with soluble eGFP. Previously, we co-transfected the channels with postsynaptic scaffold such as Homer and PSD95, which could have had a retentive effect on channel dynamics because of their documented interaction potential (40, 58, 59).

These results imply that in mature neurons pSer-1928 does not promote channel diffusion, whereas pSer-1700 reinforces the channel lateral mobility. Interestingly, the results on the double mutant revealed that the S1928E mutation was dominant on R1696Q,R1697Q. This result prompted us to investigate whether Ca_V_1.2 channels are cleaved in cultured neurons as in brain and cardiomyocytes (22–24).

### The proteolytic processing of the Ca_V_1.2 C terminus correlates with neuronal activity and depends on NMDA receptors

**Figure 5.**
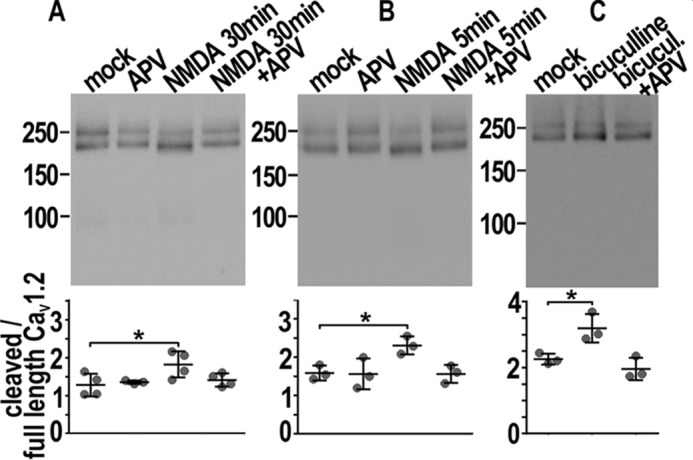
**Proteolytic processing of the Ca_V_1.2 C terminus upon activation of NMDA receptors.** *A* and *B*, immunoblots of endogenous Ca_V_1.2 in whole cell lysates from cultured neurons at DIV 10 (*A*) and 20 (*B*). Drug treatments are indicated at the *top* of each lane and were used previously (56, 57). All scatter plots show the ratio between densitometric quantification of cleaved (*lower bands*) and full-length Ca_V_1.2 (*upper bands*) in the experimental conditions corresponding to the lanes above. *A,* at DIV 10 the amount of cleaved Ca_V_1.2 increased upon 30 min of 50 μm NMDA and was inhibited by concomitant administration of dl-APV 100 μm (APV in the figure). *B,* at DIV 20 a 5-min exposure to 50 μm NMDA followed by return to drug-free medium for 30 min augmented the fraction of truncated Ca_V_1.2. Co-administration of dl-APV abolished this result. *C*, immunoblot of biotinylated surface-exposed Ca_V_1.2 extracted from mature cultured neurons treated for 1 h with vehicle (mock), bicuculline (40 μm), and bicuculline plus dl-APV (40 and 100 μm, respectively). Bicuculline increased the fraction of cleaved Ca_V_1.2 and dl-APV prevented this process. Scatter plots, mean ± S.D. *n* = 3 (*B* and *C*) to 4 (*A*), on 3–4 different cultures. Statistics: one-way ANOVA followed by Dunnett's multiple comparison test, * *p* < 0.05.

The distal C terminus of Ca_V_1.2 channels was shown to be proteolytically cleaved in brain and in cardiomyocytes (22–24). The channel cleavage is critical for phosphorylation on Ser-1700, for inhibition of channel activity, and for control of gene transcription (16, 24–27). The cleavage site is at Ser-1800 thus Ser-1700 belongs to the transmembrane core channel and Ser-1928 to the clipped distal C terminus (15). We showed that for double mutants in SPT the mobility properties of S1928E prevailed on those of R1696Q,R1697Q (Fig. 3, *E* and *F*, *yellow*). This finding led us to question whether Ca_V_1.2 is cleaved in cultured neurons. It is well known that cleavage yields a truncated transmembrane core channel of about ∼210 kDa detectable along with the full-length protein ∼240 kDa in Western blots experiments (23). It was shown that in brain cleavage, Ca_V_1.2 depends on activation of NMDA receptors (22). We determined whether these findings apply also to cultured hippocampal neurons. Western immunoblotting on neuronal lysates using an antibody directed to the channel cytoplasmic II–III loop revealed full-length and truncated channels (Fig. 5). The ratio between the intensities of the bands showed that the total amount of cleaved Ca_V_1.2 at least equaled the full-length at DIV 10 and 20 (Fig. 5, *A* and *B*, scatter plots, “mock” control). A 37% increase of cleaved Ca_V_1.2 was observed in mature neuronal cultures 30 min after a 5-min challenge with 50 μm NMDA consistent with previous findings (Fig. 5*B*) (22). Prolonged exposures to NMDA were necessary to detect a similar effect in young neurons probably because of the weaker network activity at this stage (40%, Fig. 5*A*). Both increases were prevented by the concomitant administration of the NMDAR antagonist dl-APV showing the crucial role of NMDAR for the proteolytic process (APV in Fig. 5). In line with these results, anti-Ca_V_1.2 immunoblot on neuronal lysates enriched with membrane proteins by the biotin-streptavidin binding reaction revealed that enhancing the network activity by a 1-h administration of 40 μm bicuculline increased the amount of truncated Ca_V_1.2 at the membrane by ∼45%. Concomitant administration of dl-APV abolished this result (Fig. 5*C*, scatter plots). Notably, the fraction of membrane-inserted cleaved channels exceeded by two times that of the full-length Ca_V_1.2 in basal conditions (Fig. 5*C*, *mock* control). Altogether, these results demonstrate that effective cleavage of endogenous Ca_V_1.2 correlates with increased neuronal activity and involves NMDAR. Finally, we verified whether the mutations introduced into the Ca_V_1.2 constructs allow the proteolytic processing of the C terminus. Ca_V_1.2-HA constructs expressed in HEK293 were previously shown to yield the typical two bands of full-length and truncated channels in immunoblots (28). Thus, we expressed Ca_V_1.2-HA mutants, β_1a_-eGFP, and α^2^δ-1 subunits in HEK293 and processed whole cell lysates by Western blots in the presence of dithiothreitol (DTT) to irreversibly reduce disulfide bonds and allow the optimal protein separation. All mutants were subject to cleavage (Fig. S7). The highly variable amount of the cleaved channel among experimental repetitions is likely attributable to basal functional unsteadiness of HEK293 cells (Fig. S7, table). Because of very low transfection efficiency a similar analysis could not be done in neurons.

## Discussion

In hippocampal neurons membrane-expressed Ca_V_1.2 channels were shown to distribute in clusters in which stably anchored channels exchange with diffusive extra-clustered ones to preserve a local steady-state (4, 5). Recent studies reveal the importance of selective phosphorylation on C-terminal Ser-1700 and Ser-1928 for Ca_V_1.2-mediated calcium signaling (6–9, 29). Here, by using a molecular phosphomimetic approach, we show that distinct phosphorylation at Ca_V_1.2 Ser-1700 and Ser-1928 could control channel surface trafficking and membrane expression according to the neuronal differentiation state in basal conditions. Thus, we propose that C-terminal phosphorylation at specific residues may participate in the regulation of calcium signaling by controlling the channel localization at signaling domains.

In young neurons the S1928E boosted intracellular trafficking and membrane lateral diffusion of Ca_V_1.2 without changing the overall steady-state of clusters at the membrane. Therefore, pSer-1928 probably enhances the exchange rate between clustered and diffusive channels at the surface and regulates Ca_V_1.2 internalization and/or delivery to the membrane. In contrast, in differentiated neurons S1928E promoted channel stabilization at the membrane and favored the formation of new clusters. Recent published findings show that pSer-1928 is required for Ca_V_1.2-dependent long-term potentiation and adrenergic signaling to the channel (8, 9). Another study demonstrates that pSer-1928 stimulates calcium influx and vasoconstriction in the presence of high glucose and diabetes (29). It is therefore plausible that pSer-1928 facilitates the fulfillment of the Ca_V_1.2 functions by stabilizing the channel at the membrane.

**Figure 6.**
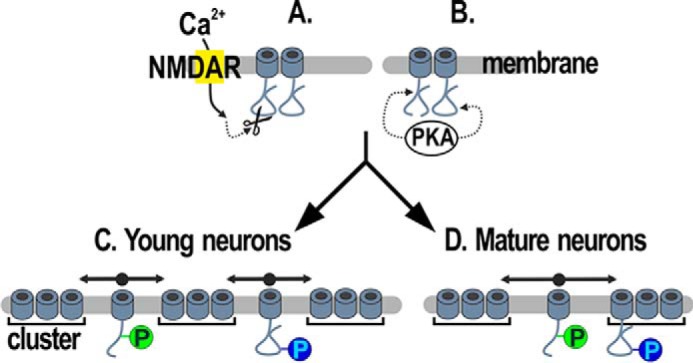
**Model of Ca_V_1.2 channels dynamics in dendrites of cultured hippocampal neurons.** Full-length and truncated channels are present in neurons. Activation of NMDA receptors (NMDAR, *yellow rectangle*) causes the cleavage of the Ca_V_1.2 C terminus (19) (*A*), which allows the PKA to phosphorylate Ser-1700 (12) (*B*). Ser-1928 is also a substrate for PKA (*B*). The majority of channels is clustered in signaling complexes. Outside of clusters, mobile channels are diffusely distributed (5). In young neurons pSer-1700 and pSer-1928 promote the diffusive behavior of extra-clustered channels (*C*). In mature neurons only pSer-1700 favors the Ca_V_1.2 diffusive state, whereas pSer-1928 stabilizes the channels at the clusters (*D*).

In young neurons R1696Q,R1697Q promoted the channel diffusive state, augmented the channel membrane levels most likely by preventing channel internalization, and increased cluster size likely by promoting a transient recruitment of channels to cluster sites. Therefore, pSer-1700 likely controls channel levels at the membrane and regulates the exchange between channel diffusive and anchored state. In mature neurons R1696Q,R1697Q still empowered the channel diffusion and probably promoted membrane expression by impairing channel internalization. Thus, pSer-1700 supports the exchange between clustered and diffusive channels and enhances channel membrane levels also in mature neurons. Finally, the trafficking properties of the double mutant resemble those of R1696Q,R1697Q in young neurons and those of S1928E in mature ones. In summary our data suggest a model (Fig. 6) in which the pSer-1700 probably favors the diffusive state in young and mature neurons (Fig. 6, *C* and *D*, *green circle*, *double sided arrows*). In stark contrast, pSer-1928 weakens channel anchoring in young neurons (*C*, *blue circle*) and stabilizes it in mature neurons also when Ser-1700 is phosphorylated (*D*, *blue circle*). Because phosphorylation on Ser-1700 is facilitated by cleavage of the C terminus (16, 25), which in turn correlates with NMDAR activity (22), we propose that the Ca_V_1.2 mobile population may be sized by concomitant activation of NMDAR and pSer-1700. Furthermore, all mutants seemed to show changes of channel internalization and/or delivery to the membrane indicating that pSer-1700 and/or pSer-1928 could play a decisive role to regulate the amount of channels at the membrane in neurons.

It is very well established that adrenergic stimulation phosphorylates Ca_V_1.2 and increases channel open probability (8, 25). This notion and our present data bring to hypothesize that channel dynamics and the clustered/non-clustered state could correlate with channel activation. Understanding this issue is essential to decisively link Ca_V_1.2 channel activity to mobility as well as subcellular localization and calcium signaling. For example, clusters of Kv2.1 were shown to contain non-conducting channels, whereas non-clustered channels were responsible for a high threshold of delayed rectifier potassium current (30). Another study showed that activity-dependent dephosphorylation releases Kv2.1 from clusters and induces a shift of the channel voltage-dependent activation revealing an important link between neuronal excitability and neurotransmission (31).

We found that phosphomimetic variants exhibited different current densities in HEK293 and neurons. In HEK293 channels interacted only with the co-transfected β_1_ and α^2^δ1. In contrast, neurons endogenously express all β subunits and three α^2^δ isoforms (32) allowing multiple subunit combinations to modulate channel activity (9, 13, 14, 33). Thus, channel regulation by auxiliary subunits is diverse in the two systems and this could explain the observed differences.

The R1696Q,R1697Q was previously shown to prevent a positive shift of voltage-dependent activation induced by the interaction between the distal and proximal C terminus (16). This regulation was not observed in our study. Two points could explain this discrepancy. First, this mechanism was characterized by co-expressing the truncated channel and the C-terminal fragment together with β_1b_ and the α^2^δ-1 subunit in tsa-201 cells, thus isolating the regulatory mechanism in a heterologous system (16). In contrast, we expressed the full-length channel in neurons and HEK293 in which only one fraction of the whole channel amount was subject to endogenous cleavage. Our electrophysiology recordings, therefore, included currents from truncated and full-length channels. Thus the regulation of the cleaved C terminus alone was impossible to isolate. Second, in neurons the channel could associate with any of the many isoforms of endogenous subunit available (32), whereas in Hulme *et al.* (16) the truncated channel interacted only with the co-transfected β_1b_ and α^2^δ-1 subunit. Essentially, the two experimental approaches are profoundly different.

It is very important to state that any experiment on cultured hippocampal neurons shows a strong culture-to-culture variability. To compensate with this, our experiments always included a control condition that was run in parallel and to which the channel mutant were compared.

We show that at least half of the whole Ca_V_1.2 population is cleaved in hippocampal cultures, the process is regulated by NMDAR activation (22) and phosphomimetic and phospho-resistant mutations preserve the proteolytic processing of the channel C terminus. Thus our data include considerable amounts of truncated and full-length channels. We have previously shown that in mature neurons Ca_V_1.2 clusters were unaltered or rather slightly increased upon direct activation of NMDAR (12). Furthermore, KCl depolarization, which activates also NMDAR, did not affect the dynamics of Ca_V_1.2 in mature neurons and only slightly increased it in young ones (Ref. 5, but also see Ref. 17). Thus, channel cleavage itself unlikely explains our present data. Furthermore, we found that about 2/3 of membrane-expressed Ca_V_1.2 are cleaved meaning that truncated channels were highly represented in our SPT results. Intriguingly, the channel lateral mobility of R1696Q,R1697Q,S1928E mutants was strongly influenced by S1928E, which is within the clipped C terminus (15). In the case of full-length Ca_V_1.2 this is explained by the channel integrity. For cleaved Ca_V_1.2 this finding implies that the clipped C terminus associates to the main channel although the R1696Q,R1697Q prevents the interaction between proximal and distal C terminus. This could be achieved via intramolecular interactions involving the channel cytoplasmic regions and the distal C terminus (34, 35) and/or by the physical coupling with the AKAP79/150 scaffold, which simultaneously interacts with Ca_V_1.2 N terminus, the I-II loop, and the leucine zipper domain, which is downstream of Ser-1928 (36). Although speculative here, the association of the clipped C terminus with the main channel is not surprising because of the importance of pSer-1928 for the neuronal physiology (6–9).

It has been widely shown that a potentiated dynamics of membrane proteins parallels a diminished efficacy of establishing retentive interactions at signaling domains (19–21). We persistently observed clustered distribution of Ca_V_1.2 and a population of confined and stable channels, consistent with cluster localization (5), in all conditions in which channel mobility increased. Thus, not all stabilizing interactions were equally affected by the mutations meaning that multiple concomitant mechanisms may participate to channel anchoring. Finally, the same mutations determined different channel dynamic states and/or distribution in young and mature neurons. This implies permissive anchoring modes and, therefore, suggests a differentiation dependent switch of Ca_V_1.2 stabilizing interactions. Several scaffolding proteins including AKAP79/150, AKAP15/18, PDZ domain containing proteins, and α-actinin were shown to directly interact with Ca_V_1.2 in neurons, modulate their trafficking, and tether signaling transducers to the close proximity of the channels (6, 34, 36–40). Future studies are necessary to examine how phosphorylation and channel cleavage affect the channel binding with these putative scaffolds.

In conclusion, our results unravel a previously unknown mechanism by which selective phosphorylation at Ser-1700 and Ser-1928 differentially regulates the surface traffic of Ca_V_1.2. Dysregulation of this mechanism could impair channel steady-state levels upstream of Ca_V_1.2-mediated signaling pathways. These findings provide the basis for future mechanistic studies on the surface localization of Ca_V_1.2 under physiological and pathological conditions where channel phosphorylation and intracellular calcium signaling are involved.

## Experimental procedures

### Primary cultures of mouse and rat hippocampal neurons and transfection

Low-density cultures of hippocampal neurons were prepared from 16.5-day-old embryonic BALB/c mice or from 18-day-old embryonic Sprague-Dawley rats of either sex as described previously (4, 41). Briefly, dissected hippocampi were dissociated by 2.5% trypsin and trituration. Isolated neurons were plated on poly-l-lysine-coated glass coverslips at a density of 3500 cells/cm^2^ or 100–200 × 10^3^ cells/ml for mice and rat cultures, respectively. After 4 h from plating, coverslips were transferred neuron side-down into a 60-mm culture dish with a glial feeder layer. Neurons and glial feeder layer were cultured in serum-free Neurobasal medium (Invitrogen) supplemented with GlutaMax and B27 (Invitrogen). All experiments with the exception of single tracking experiments were performed on mouse neurons. Murine neurons were transfected with Lipofectamine 2000 (Invitrogen) and rat neurons with the calcium phosphate method (42).

### Analysis of phosphorylation status of Ca_V_1.2 Ser-1700 and Ser-1928

Hippocampal cultures grown in 60-mm Petri dishes were homogenized in 0.5 ml of 1% Triton X-100, 10 mm Tris-Cl, pH 7.4, 20 mm EDTA, 10 mm EGTA containing protease inhibitors (1 μg/ml of pepstatin A, 10 μg/ml of leupeptin, 20 μg/ml of aprotinin, 200 nm phenylmethanesulfonyl fluoride) and phosphatase inhibitors (1 mm *p-*nitrophenyl phosphate and 4 μm microcystin LR), which effectively prevent dephosphorylation of Ca_V_1.2 (43, 44). Triton X-100-insoluble material was removed by ultracentrifugation (240,000 × *g*, 30 min). Ca_V_1.2 was immunoprecipitated with 4 μg of our FP1 antibody and washed three times with TBS containing 0.1% Triton X-100 (45). To stoichiometrically phosphorylate Ca_V_1.2 *in vitro* (43, 46) immunocomplexes were incubated in 50 μl of phosphorylation buffer (0.1% Triton X-100, 50 mm HEPES-NaOH, pH 7.4, 10 mm MgCl_2_, 0.5 mm EGTA, 50 μm ATP, 0.5 mm DTT, 1 μg/ml of pepstatin A, 10 μg/ml of leupeptin, 20 μg/ml of aprotinin) containing 0.5–1 μg of catalytically active purified catalytic subunit of PKA (Sigma) in a thermomixer for 30 min at 32 °C. Immunocomplexes were washed three times with 10 mm Tris-HCl, pH 7.4, 75 mm NaCl, 20 mm EDTA, 10 mm EGTA, 20 mm sodium pyrophosphate, 50 mm NaF, 20 mm 2-glycerolphosphate, 1 mm *p-*nitrophenyl phosphate, and once with 10 mm Tris-HCl, pH 7.4, before immunoblotting with anti-pSer-1928 (46), reprobing with anti-pSer-1700 (15) and subsequently with FP1 (for more details see Refs. 43 and 47). All immunoblots were quantified by densitometry of the film exposures (22). To quantify phosphorylation levels of Ser-1700 both upper and lower bands were used.

### Culture and transfection of HEK293 cells

Cells were grown at 37 °C and 5% CO_2_ in DMEM plus 10% fetal bovine serum, 1% penicillin/streptomycin, and 1% GlutaMAX (all GIBCO). Cells were co-transfected at 70% confluence with untagged, HA- or eGFP-tagged Ca_V_1.2 constructs together with pβA-α^2^δ1 and pβA-β_1a_-eGFP (molar ratio 1:1:1) using Lipofectamine 2000 (Invitrogen). Western blotting was performed 48 h after transfection. For electrophysiology experiments cells were re-plated at low density 24 h after transfection and recorded 24 h later.

### Plasmids

Generation of pβA-eGFP and pβA-Ca_V_1.2-HA (rat neuronal Ca_V_1.2: GenBank^TM^ accession number M67515) were previously described (4, 37). The following mutations were introduced by SOE-PCR: (i) dihydropyridine-insensitive T1039Y (48–50); (ii) phosphomimetic S1928E, R1696Q,R1697Q, R1696Q,R1697Q-SE, S1700E, S1700E,S1928E; (iii) and phospho-resistant S1700A, S1928A, S1700A,S1928A. Briefly, cDNA sequences of Ca_V_1.2 were PCR amplified with overlapping primers introducing the mutations of interest in separate PCR using pβA-Ca_V_1.2 as template. The two PCR products were then used as templates for a final PCR with flanking primers to connect the nucleotide sequences. The fusion fragment containing the T1039Y mutation was finally SpeI/EcoRV-digested and ligated into the corresponding sites of pβA-Ca_V_1.2, yielding pβA-Ca_V_1.2^DHP−^. The fusion fragments containing the phosphomimetic and phospho-resistant mutations were digested with SbfI/SacI and ligated into the corresponding sites of pβA-Ca_V_1.2-HA or pβA-eGFP-Ca_V_1.2, yielding pβA-Ca_V_1.2-HA-SE, pβA-Ca_V_1.2-HA-RRQQ, pβA-Ca_V_1.2-HA-RRQQ-SE, pβA-Ca_V_1.2-HA-S1928A, pβA-Ca_V_1.2-HA-S1700A, pβA-Ca_V_1.2-HA-S1700A,S1928A or pβA-eGFP-Ca_V_1.2-SE, pβA-eGFP-Ca_V_1.2-RRQQ, pβA-eGFP-Ca_V_1.2-RRQQ-SE, pβA-eGFP-Ca_V_1.2-S1928A, pβA-eGFP-Ca_V_1.2-S1700A, pβA-eGFP-Ca_V_1.2-S1700A,S1928A. The phosphomimetic S1928E, R1696Q,R1697Q, and R1696Q,R1697Q-SE fusion fragments were digested with SacI/SfbI then ligated into the corresponding sites of pβA-Ca_V_1.2*^DHP^*^−^ and pβA-Ca_V_1.2, yielding pβA-Ca_V_1.2-SE*^DHP^*^−^, pβA-Ca_V_1.2-RRQQ*^DHP^*^−^, pβA-Ca_V_1.2-RRQQ-SE*^DHP^*^−^, pβA-Ca_V_1.2-SE, pβA-Ca_V_1.2-RRQQ, pβA-Ca_V_1.2-RRQQ-SE.

To generate the pβA-β_1a_-eGFP, the rabbit β_1a_ (GenBank^TM^ number M25514) was isolated from pβA-β_1a_-V5 (13) by NdeI/BglII digest and cloned in the respective sites of pβA-β_2a_-eGFP (18). Sequence integrity of all the newly generated constructs was confirmed by sequencing (Eurofins, Austria).

### Surface immunolabeling of HA-tagged Ca_V_1.2 constructs and image acquisition

For surface immunolabeling experiments (12) living neurons were incubated with the rat anti-HA antibody (1:100) (Roche Applied Sciece) for 30 min at 37 °C in conditioned medium, quickly washed, and fixed with 4% paraformaldehyde, 4% sucrose in phosphate buffer at room temperature for 5 min. After fixation, neurons were washed with PBS for 30 min, blocked with 5% goat serum and 0.2% BSA for 30 min, and labeled with anti-rat Alexa Fluor 594 (1:4000, 1 h). Dynasore (80 μm) (Invitrogen) was provided together with the anti-HA during incubation with the primary anti-HA antibody (5). Coverslips were mounted in *p-*phenylenediamine glycerol to retard photobleaching and observed with a conventional epifluorescence microscope using a ×60, Apochromat 1.42 NA oil-immersion objective lens. Images were acquired with a cooled CCD camera (SPOT; Diagnostic Instruments). Dendritic branches between 50 and 100 μm from the soma were placed at the central region of the image. Image acquisition was conducted blindly.

### Quantification of Ca_V_1.2-HA clusters and morphology of dendrites

Fourteen-bit gray-scale images of anti-HA (red channel) and eGFP (green channel) were acquired and analyzed as described previously (5, 12). Briefly, corresponding images were aligned and the eGFP image was used to select one dendritic segment about 20–30-μm long for analysis. Using the eGFP image as reference, ROIs were drawn around the shaft and the adjacent regions containing the spines. The shaft area was measured and the number of spines was counted. The Ca_V_1.2-HA image was background-flattened (MetaMorph software, Universal Imaging) and thresholded to trace the fluorescent clusters as accurately as possible. The number of clusters and their average gray values were quantified using the integrated morphometric analysis option of MetaMorph. Cluster density was calculated as the ratio between the number of clusters in the shaft and shaft area (density in the shafts) or number of spines (density in the spines) for each analyzed dendritic segment. Cluster intensity was calculated by subtracting the background from the cluster average gray values. Values of densities and intensities were averaged separately for shafts and spines for each dendritic segment to obtain only one value per parameter for each analyzed neuron. Values of cluster density and intensity of HA-tagged Ca_V_1.2 constructs were expressed as the percentage of control. Thickness of dendrites and spine frequency were obtained as the shaft area/dendritic length and number of spines/dendritic length, respectively. All surface expression experiments and analysis were conducted blindly.

### FRAP analysis

FRAP was performed on 9–11 (DIV 10) or 19–22 (DIV 20) day-old cultured neurons transfected with pβA-eGFP-Ca_V_1.2, pβA-eGFP-Ca_V_1.2-SE, pβA-eGFP-Ca_V_1.2-RRQQ on a conventional epifluorescence microscope equipped with a ×60, 1.42 NA oil immersion lens (Olympus), at 37 °C. Neurons were imaged in Tyrode's physiological solution containing (in mm): 130 NaCl, 2.5 KCl, 2 CaCl_2_, 2 MgCl_2_, 10 HEPES, 30 glucose. Fluorescence was excited using the 488 nm line of a LED pE-2 lamp (CoolLed) or a Sola Light Engines (Lumencor). Imaging frequency in the pre-bleach and post-bleach phases was set at 2 Hz. Four round ROIs were placed along dendritic shafts and spines when possible. Bleaching was achieved by rapid 405 laser pulses (Visitron Systems, Germany). FRAP experiments were analyzed as before (5). Average fluorescence in ROIs was measured, background subtracted, corrected for overall photobleaching in each time frame, double normalized so that the pre-bleach intensity was set to 1 and the first frame after photobleaching to 0, and finally plotted as a function of time. Amplitude was calculated by averaging the last five time points of individual normalized curves. Fluorescence intensity was measured using MetaMorph software. FRAP experiments were performed blindly.

### Single particle tracking

To label the Ca_V_1.2-HA channel mutants, anti-rat quantum dots with 655 nm fluorescence emission (anti-rat QD-655) were prepared as follows: QD-655 nm fluorescence emission (highly cross-adsorbed goat F(ab′)_2_ anti-rat IgG conjugate (heavy + light chains), 0.1 μm, Invitrogen) contained in 1 μl of the commercially available suspension were pre-coated with mouse anti-rat (0.5 μg) in 10 μl of PBS for 30 min and blocked with casein (1-fold dilution, Vector Labs) for 15 min. Transfected neurons were incubated with anti-HA (Roche, 1:50) for 15 min followed by a 5-min incubation with goat F(ab′)_2_ anti-rat QD-655 (1:2000, Invitrogen) in cultured medium at 37 °C. After washing, cells were mounted in an open chamber and used for imaging experiments for up to 20 min at 37 °C. Time lapse recordings of QD-labeled Ca_V_1.2-HA relative to eGFP fluorescence were acquired at 37 °C in an open chamber mounted onto an inverted microscope (Axio Observer, Zeiss) equipped with a ×100/1.3 NA objective and a back-illuminated CCD camera (iXon Ultra 897, Andor). Emitted fluorescence was acquired through HQ560/80M and 655WB20 filters, respectively. Time lapse recordings of QDs were obtained with an integration time of 30 ms with up to 1000–2000 consecutive frames. QD fixed to the coverslip allowed compensation for mechanical drifts of the stage.

### Channel tracking and analysis

The tracking of HA-tagged Ca_V_1.2 channel mutants using QDs was performed by localizing single quantum dots in the image plane by a custom-made software based on MATHLAB (MathWorks) (51). Single QDs were identified based on their fluorescent properties showing on/off emission as an indicator for a single QD. QD fluorescence profiles were fitted with a two-dimensional Gaussian function. The localization accuracy was between 30 and 50 nm, calculated from the offset of MSD-plots over time intervals from fixed QDs on coverslips. This empirical method takes into account the motion artifact of the stage as well as the light condition within experiments. The labeling density was adjusted to allow the use of a closest neighbor reconnection algorithm, taking the blinking properties into calculation. Trajectories were reconnected if they matched with the following parameters: first, the displacement of the QDs between two frames had to be less than two pixels to be assigned to the trajectory. Second, off-periods due to blinking had to be shorter than 20 frames (time points). Reconstructed trajectories, particular trajectories longer than 100 frames, showed different modes of diffusion. The diffusion profile was accessed by the use of a sliding MSD calculation for trajectories longer than 100 frames, starting with the initial 20 time points. Each time point was used to start a new MSD calculation and the slope of a linear fit through the first 4 points of the MSD were used to calculate the diffusion coefficient. Based on the tracking of fixed QDs motion artifacts of the microscope were calculated and particles less mobile than 0.001 μm^2^/s are judged as immobile. Changes of the mode of diffusion were from free Brownian to confined (52). In brief, a molecule is considered as confined if the explored surface for a given time interval is smaller as expected for free Brownian diffusion in the membrane.

### Electrophysiology

Barium currents through Ca_V_1.2^DHP−^ channels were recorded using the whole-cell patch clamp technique. Patch pipettes were pulled from borosilicate glass (Harvard Apparatus), fire-polished (Microforge MF-830, Narishige), and had resistances of 2.5–4 MΩ when filled with the following (in mm): 120 cesium methanesulfonate, 1 MgCl_2_, 0.1 CaCl_2_, 10 HEPES, 0.5 EGTA, 2 MgATP, 0.3 NaGTP (pH 7.2 with CsOH). The bath solution contained the following (in mm): 10 BaCl_2_, 110 NaCl, 20 TEA-Cl, 5 4-AP, 10 HEPES, 2 MgCl_2_, 3 KCl, 10 glucose, 0.001 Triton X-100 (pH 7.4 with NaOH). Currents were recorded with an EPC 10 amplifier controlled by PatchMaster software (HEKA Elektronik Dr. Schulze GmbH, Germany). Linear leak, series resistance, and capacitive currents were digitally compensated. Neurons were held at −50 mV to inactivate current through T-type calcium channels, all other endogenous calcium channels were blocked with the following antagonists (53, 54): 800 nm ω-Agatoxin IVA, 3 μm ω-Conotoxin GVIA, 3 μm ω-conotoxin MVIIC, 1 μm SNX-482 (all from Alomone), 30 μm nifedipine (Sigma).

In HEK293 cells barium currents through Ca_V_1.2 channel constructs were recorded in whole-cell configuration of the patch clamp technique using the amplifier List L/M-EPC 7 (LIST Electronics) and the A/D − D/A converter Digidata 1200 (Molecular Devices). The pCLAMP software (Molecular Devices) was used for data acquisition and analyses. When filled with internal solution (composition in mm: 114 CsCl, 10 EGTA, 10 HEPES, 5 MgATP, adjusted to pH 7.2 with CsOH) the patch–electrodes had a tip resistance of 2–3 MΩ. The external solution contained (in mm): 150 choline-Cl, 15 BaCl_2_, 10 HEPES, 1 MgCl_2_, adjusted to pH 7.4 with CsOH. Cell membrane capacitance was determined by integration of the capacitive transient elicited by a 10-mV hyperpolarizing step from −50 mV. Ion currents were normalized to cell membrane capacitance and expressed as pA/pF. The current-voltage dependence was fitted according to equation,

(Eq. 1) $$I\;=\;G_{\max}\;\cdot \;\left(V-V_{\text{rev}}\right)/\left(1\;+\;\exp\left(-\left(V-V_{1/2}\right)/k\right)\right)$$

where *G*_max_ is the maximum conductance of the Ca_V_1.2 calcium channels, *V*_rev_ is the extrapolated reversal potential of the calcium current, *V*_1/2_ is the potential for half-maximal conductance, and *k* is the slope.

### Western blot on treated cultured neurons and HEK293 cells

Dissociated hippocampal neurons from 16.5-day-old embryonic BALB/c mice were plated on poly-l-lysine (1 mg/ml)-coated wells (20 mm) at the density of 150,000 cells per well. Young neurons were treated for 30 min with NMDA (50 μm, Sigma), dl-APV (100 μm, Sigma), or NMDA plus dl-APV (50 and 100 μm, respectively) in conditioned medium. Neurons at DIV 20 were treated for 5 min with 50 μm NMDA and then returned to conditioned medium for 30 min. When necessary, neurons were pre-treated with dl-APV before concomitant administration of NMDA and dl-APV to block NMDA receptors first. To equalize the handling procedures, the other experimental conditions were preceded by sham conditions. After treatments, neurons were washed using cold Hank's balanced salt solution and lysated with ice-cold bRIA buffer modified from Hulme *et al.* (55): 50 mm Tris-HCl, pH 7.4, 150 mm NaCl, 10 mm EDTA, 1% Triton X-100, protease inhibitors mixture (Sigma, P8340). Lysates were centrifuged at 16,000 × *g* for 10 min at 4 °C to remove the insolubilized material. Protein concentration of lysates was determined using BCA Kit assay (Pierce). 10 μg of protein/well were loaded onto a 7.5% acrylamide-bisacrylamide gel. Proteins were blotted to nitrocellulose membranes (Bio-Rad, 162-0115) at 80 V for 120 min in ice. After 1 h incubation with blocking buffer (20 mm Tris, pH 7.4, 150 mm NaCl, 0.1% Tween 20, and 4% dried nonfat milk (Regilait)) at room temperature, membranes were exposed to rabbit anti-Ca_V_1.2 (Alomone, ACC-3300 (28)) (1:500) in blocking buffer overnight at 4 °C. Secondary antibody was applied for 1 h in blocking buffer at room temperature (anti-rabbit IgG peroxidase conjugate, 1:4000; Sigma, A8275). The chemiluminescent signal was developed with an ECL detection system (GE Healthcare) and detected using ChemiDoc^TM^ MP System (Bio-Rad). The intensity of non-saturated band signals was quantified using ImageLab software (Bio-Rad). The levels of endogenous cleaved *versus* full-length Ca_V_1.2 were expressed as ratio between the intensities of the corresponding bands. The lysis buffer used for HEK293 cells contained 10 mm Tris-HCl, pH 7.4, 1% Triton X-100, 10 mm DTT and protease inhibitor mixture. Subsequently, lysates were processed as indicated above.

### Surface biotinylation assay

Neurons were plated as for Western blot procedures. Bicuculline (40 μm) (Sigma) and dl-APV (100 μm) (Sigma) were administrated for 1 h. Controls were treated with vehicle solution. Treated neurons were washed twice with PBS at pH 8 containing 0.1 mm CaCl_2_, 1.0 mm MgCl_2_, incubated with EZ-Link Sulfo-NHS-LC-Biotine (0.5 mg/ml in PBS, Thermo Scientific) for 5 min at 37 °C, washed once with TBS (20 mm Tris, 150 mm NaCl, pH 8) supplemented with 0.1 mm CaCl_2_, 1.0 mm MgCl_2_, and 50 mm glycine at room temperature, and twice with TBS plus 0.1 mm CaCl_2_ and 1.0 mm MgCl_2_ on ice. After extraction using bRIA-modified buffer (see above), 200 μg (BCA Kit assay, Pierce) of proteins from each condition were incubated overnight at 4 °C with streptavidin-conjugated beads (Pierce). After incubation, beads were washed three times with bRIA-modified buffer by centrifugation at 5000 × *g* for 5 min at 4 °C. Bead-conjugated membrane proteins were resuspended in sample buffer 3 times (350 mm Tris-HCl, pH 6.8, 10% glycerol, 1% SDS, 10% β-mercaptoethanol, 0.1% bromphenol blue) and loaded onto a 7.5% acrylamide-bisacrylamide gel. Membranes were then processed as described above.

### Statistical analysis

Statistical significance between two groups was calculated with Mann-Whitney rank sum test when the Shapiro-Wilk Normality test failed (Sigma plot 13.0). Multiple comparison test among the populations was performed using one-way ANOVA followed by a Dunn's post or Tukey post hoc test as indicated in the text (GraphPad Prism 7). Time point statistics of FRAP experiments was performed using the built-in data analysis plug-in in Excel. Graphs and figures were generated using Origin 7.5, Adobe Photoshop CS6, and CorelDraw X7 software.

## Author contributions

A. S., C. R., A. F., and M. C. generated the channel constructs; A. F. and V. D. B. conducted and analyzed imaging experiments; A. F. performed and analyzed the biochemical experiments on channel cleavage; R. S. and G. J. O. performed and analyzed the electrophysiology on neurons and S. S. and B. P. on HEK293 cells; A. F., M. H., and V. D. B. conducted and analyzed SPT experiments; B. L. and J. W. H. performed and analyzed Ca_V_1.2 phosphorylation assays; A. F. and V. D. B. designed experiments; J. W. H. wrote the results, figure legends, and material and methods of the Western blot analysis of endogenous Ca_V_1.2 phosphorylation; V. D. B. conceived, coordinated the study, and wrote the paper. All authors reviewed the results and edited and approved the final version of the manuscript.

## Supplementary Material

Supporting Information

## Footnotes

5 The abbreviations used are:

DIV: days *in vitro*
ANOVA: analysis of variance
eGFP: enhanced green fluorescent protein
FRAP: fluorescence recovery after photobleaching
SPT: single particle tracking
QD: quantum dots
MSD: mean squared displacement
ROI: region of interest
dl-APV: dl-2-amino-5-phosphonopentanoic.
