# Supplementary material for: Molecular mimicking of C-terminal phosphorylation tunes the surface dynamics of CaV1.2 calcium channels in hippocampal neurons
Source: J Biol Chem. 2017 Nov 27;293(3):1040–53. doi: 10.1074/jbc.M117.799585 (PMC5777246; doi:10.1074/jbc.M117.799585)
Supplement: Supporting Information [file supp_293_3_1040__index.html]

Molecular mimicking of C-terminal phosphorylation tunes the surface dynamics of CaV1.2 calcium channels in hippocampal neurons. — Molecular mimicking of C-terminal phosphorylation tunes the surface dynamics of CaV1.2 calcium channels in hippocampal neurons — Phosphorylation dependent dynamics of CaV1.2 — Supporting Information 

# Molecular mimicking of C-terminal phosphorylation tunes the surface dynamics of CaV1.2 calcium channels in hippocampal neurons

## Supporting Information

- Supplemental data (.pdf, 609 KB) - Figures and tables
