# Supplementary material for: Molecular mimicking of C-terminal phosphorylation tunes the surface dynamics of CaV1.2 calcium channels in hippocampal neurons
Source: J Biol Chem. 2017 Nov 27;293(3):1040–53. doi: 10.1074/jbc.M117.799585 (PMC5777246; doi:10.1074/jbc.M117.799585)
Supplement: Supporting Information [file 10.1074_M117.799585_jbc.M117.799585-1.pdf]

Molecular mimicking of C-terminal phosphorylation tunes the surface dynamics of Ca_V1.2 calcium channels in hippocampal neurons.

Alessandra Folci, Angela Steinberger, Boram Lee, Ruslan Stanika, Susanne Scheruebel, Marta Campiglio, Claudia Ramprecht, Brigitte Pelzmann, Johannes W. Hell, Gerald J. Obermair, Martin Heine, and Valentina Di Biase.

Supplemental Fig S1
Supplemental Fig S2
Supplemental Fig S3
Supplemental Fig S4
Supplemental Fig S5
Supplemental Fig S6
Supplemental Fig S7
Table S1

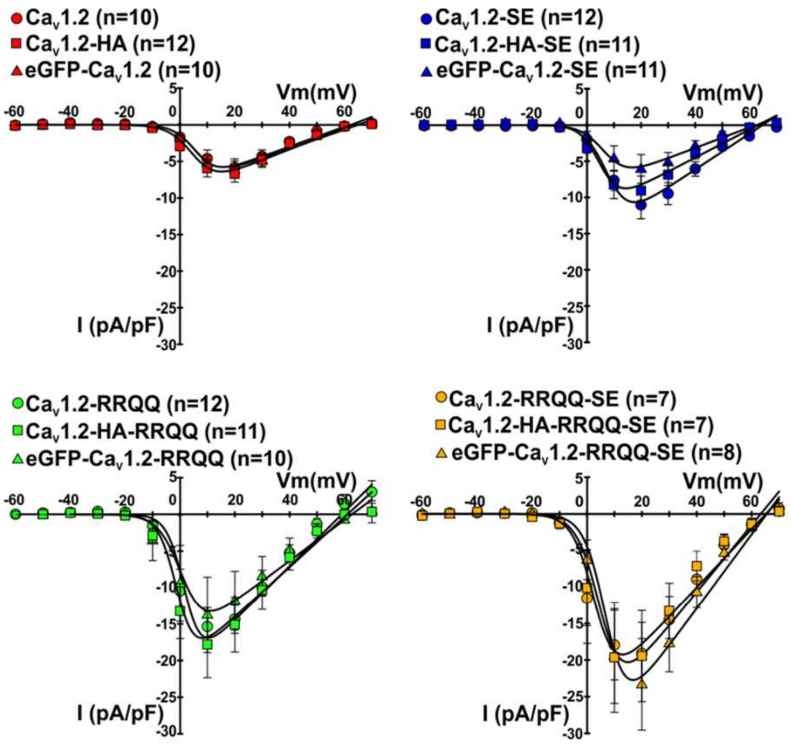

Construct	G_{max} (nS/pF)	V_{rev} (mV)	$V_{1/2}$ (mV)	k (mV)
Ca$_V$1.2	0.13±0.03	65.05±4.19	6.74±0.90	4.14±0.17
Ca$_V$1.2-HA	0.15±0.02	62.70±2.11	5.31±0.83	4.33±0.37
eGFP-Ca$_V$1.2	0.14±0.02	61.94±1.27	6.27±0.78	3.93±0.21
Ca$_V$1.2-SE	0.25±0.06	64.86±2.11	8.01±0.83	4.13±0.30
Ca$_V$1.2-HA-SE	0.19±0.05	63.31±2.03	5.23±1.00	3.65±0.37
eGFP-Ca$_V$1.2-SE	0.14±0.03	62.39±1.47	7.12±0.38	4.23±0.15
Ca$_V$1.2-RRQQ	0.37±0.06	59.06±1.42	1.88±1.69	3.16±0.43
Ca$_V$1.2-HA-RRQQ	0.34±0.07	61.08±2.89	-0.88±1.32	3.33±0.23
eGFP-Ca$_V$1.2-RRQQ	0.28±0.10	62.73±2.00	1.04±2.08	4.17±0.32
Ca$_V$1.2-RRQQ-SE	0.46±0.10	63.32±1.91	5.13±2.20	4.11±0.40
Ca$_V$1.2-HA-RRQQ-SE	0.40±0.13	65.19±3.81	3.13±0.95	3.94±0.37
eGFP-Ca$_V$1.2-RRQQ-SE	0.51±0.13	65.50±0.92	7.29±1.43	3.88±0.19

Construct	I_{max} (pA/pF)
Ca$_V$1.2	-5.86±1.21
Ca$_V$1.2-SE	-11.14±2.63
Ca$_V$1.2-RRQQ	-16.62±2.63**
Ca$_V$1.2-RRQQ-SE	-22.65±5.82##

Statistics: Kruskal-Wallis Test followed by Dunn-Bonferroni pairwise comparison post hoc analysis.
Table on the left: HA- and eGFP-tagged versus untagged channel constructs for G_{max}, V_{rev}, $V_{1/2}$, and k; n.s. p>0.05
Table on the right: **, *p*=0.007; ##, *p*=0.006

Fig. S1. Density-to-voltage relationship of Ba^{2+} currents through untagged (circles), HA- (squares) and eGFP-tagged (triangles) Ca$_V$1.2 (red), Ca$_V$1.2-S1928E (blue), Ca$_V$1.2-RRQQ (green), and Ca$_V$1.2-RRQQ-SE (yellow) constructs in HEK293 cells. The HA tag is inserted into the extracellular loop after the transmembrane helix IIS5 whereas the eGFP is fused to the Ca$_V$1.2 N-terminus. The effect of the HA and eGFP tags were tested to exclude an impairment of channel currents. HEK293 cells were co-transfected with the Ca$_V$1.2 channel constructs and the auxiliary $\alpha 2\delta$–1 and eGFP tagged β_{1a} subunits at equimolar ratio. In eGFP positive cells Ba^{2+} currents were elicited by applying 10 mV incremental depolarizing voltage steps from -60mV to +70 mV for 800 ms. The figure shows that the I-V plot for each tagged phospho-mimetic Ca$_V$1.2 constructs is similar to the corresponding untagged pair. No statistical difference was found for G_{max}, V_{rev}, $V_{1/2,}$ and k (Table, left) values of untagged channel constructs compared to the HA- or eGFP-tagged pair carrying the same mutation, indicating that the eGFP and HA tags do not modify channel current properties. Statistics: Kruskal-Wallis Test followed by Dunn-Bonferroni post-hoc analysis; n.s., $p > 0.05$. Current density (I_{max}, Table, right) increased for the Ca$_V$1.2-RRQQ and Ca$_V$1.2-RRQQ-SE consistent with release of the constitutive auto-inhibitory interaction between the proximal and distal C-terminus induced by RR1696-1697QQ mutations (15). Statistics: Kruskal-Wallis Test followed by Dunn-Bonferroni post-hoc analysis; **, $p = 0.007$; ##, $p = 0.006$. Mean ± SEM; n is indicated in parenthesis.

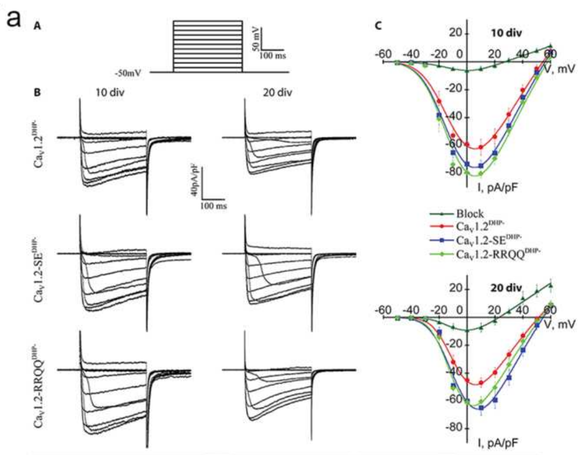

	DIV10			DIV20		
	I_{max}, pA/pF	$V_{1/2}$, mV	n	I_{max}, pA/pF	$V_{1/2}$, mV	n
$Ca_V1.2^{DHP-}$	-61.3±5.6	-9.4±1.7	13	-46.8±3.2	-7.0±1.4	15
$Ca_V1.2\text{-}SE^{DHP-}$	-74.6±4.0*	-12.2±1.9	14	-64.7±5.5**	-6.5±1.7	16
$Ca_V1.2\text{-}RRQQ^{DHP-}$	-80.2±3.2**	-12.1±1.5	12	-60.4±3.7*	-9.7±1.6	14

Statistics: ANOVA with Holm-Sidak posthoc analysis.
DIV 10: I_{max}, $F_{2,38}$=4.7; p=0.016, $V_{1/2}F_{2,38}$=0.9*, p=0.44;
DIV 20, I_{max}, $F_{2,44}$=4.6; p=0.015, $V_{1/2}F_{2,38}$=1.1, p=0.34 (*p<0.05, **p<0.01).

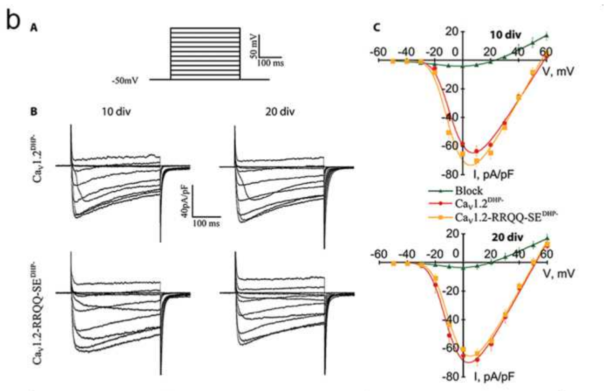

	DIV10			DIV20		
	I_{max}, pA/pF	$V_{1/2}$, mV	n	I_{max}, pA/pF	$V_{1/2}$, mV	n
$Ca_V1.2^{DHP-}$	-63.5±3.4	-5.6±1.2	13	-67.8±4.1	-8.0±1.7	13
$Ca_V1.2\text{-}RRQQ\text{-}SE^{DHP-}$	-70.2±4.1	-6.2±1.2	12	-63.5±3.2	-6.3±1.6	14

Statistics: T-test: DIV 10: I_{max}, t(23)=-1.271, p=0.216; $V_{1/2}$, t(23)=-0.358, p=0.723;
DIV 20, I_{max}, t(25)=-0.854, p=0.401; $V_{1/2}$, t(25)=-0.716, p=0.481.

Fig. S2. Using Ba^{2+} as charge carrier we examined the functional effect of the S1928E, R1696-1697Q, and R1696-1697Q-S1928E mutations on $Ca_V1.2$. Neurons exogenously expressed $Ca_V1.2^{DHP-}$, $Ca_V1.2\text{-}SE^{DHP-}$, $Ca_V1.2\text{-}RRQQ^{DHP-}$, $Ca_V1.2\text{-}RRQQ\text{-}SE^{DHP-}$ rendered insensitive to dihydropyridines by a T1039Y mutation (48-50). A cocktail of toxins including 800 nM ω-Agatoxin IVA, 3 μM ω-Conotoxin GVIA, 3 μM ω-conotoxin MVIIC, 1 μM SNX-482, 30 μM nifedipine was administrated to neurons to silence the whole endogenous population of voltage gated calcium channels (dark green). *(a. A*) 300 ms step pulses voltage between -50 mV and 60 mV in 10 mV increments elicited barium currents via $Ca_V1.2^{DHP-}$, $Ca_V1.2\text{-}SE^{DHP-}$, $Ca_V1.2\text{-}RRQQ^{DHP-}$, and *(b. A)* $Ca_V1.2\text{-}RRQQ\text{-}SE^{DHP-}$ (a. and b., *B*). Representative current traces for all constructs in young (DIV10) and mature neurons (DIV 20). (a., *C*) Current voltage relationship in neurons expressing $Ca_V1.2^{DHP-}$(red), $Ca_V1.2\text{-}SE^{DHP-}$ (blue), or $Ca_V1.2\text{-}RRQQ^{DHP-}$ (light green) and eGFP (green) at DIV 10 and 20 show a statistically significant increase of current densities in mutated constructs compared to wild type Cav1.2. Instead, $Ca_V1.2\text{-}RRQQ\text{-}SE^{DHP-}$ was comparable to controls in young and mature neurons.

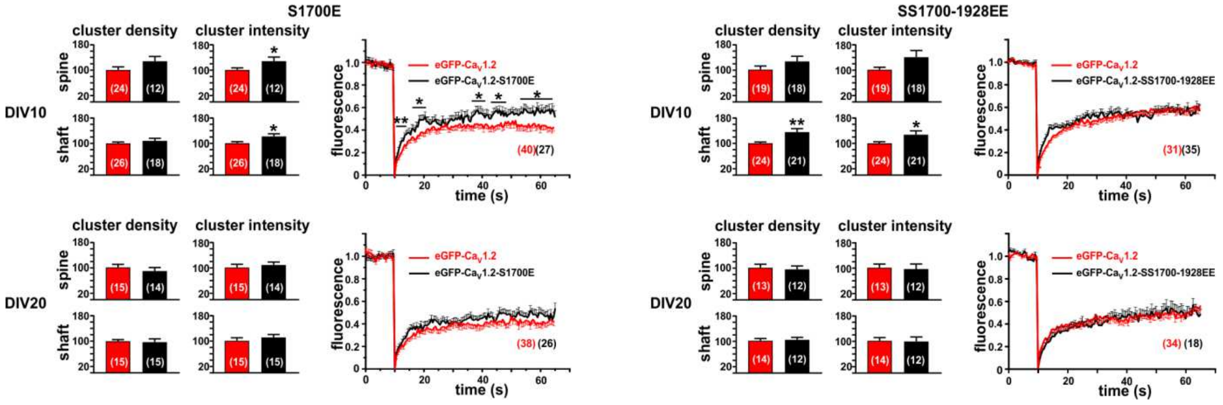

Fig. S3. Quantification of membrane expression and dynamics of channels with S1700E and SS1700-1928EE substitutions. Quantitative analysis of surface expressed clusters of Ca_V1.2-HA-S1700E (black) and Ca_V1.2-HA-SS1700-1928EE (black) versus control Ca_V1.2-HA (red) revealed results comparable to those of the RRQQ (see Fig. 2 in the text). Bar graphs, mean ± SEM; number of analyzed dendritic segments each from a different neuron is in parenthesis. Similarly the trend of eGFP-Ca_V1.2-S1700E and eGFP-Ca_V1.2-SS1700-1928EE compares to that of the RRQQ mutants (see Fig. 3 in the text). Statistics: t-test, *, $0.01<p<0.5$; **, $0.001<p<0.01$.

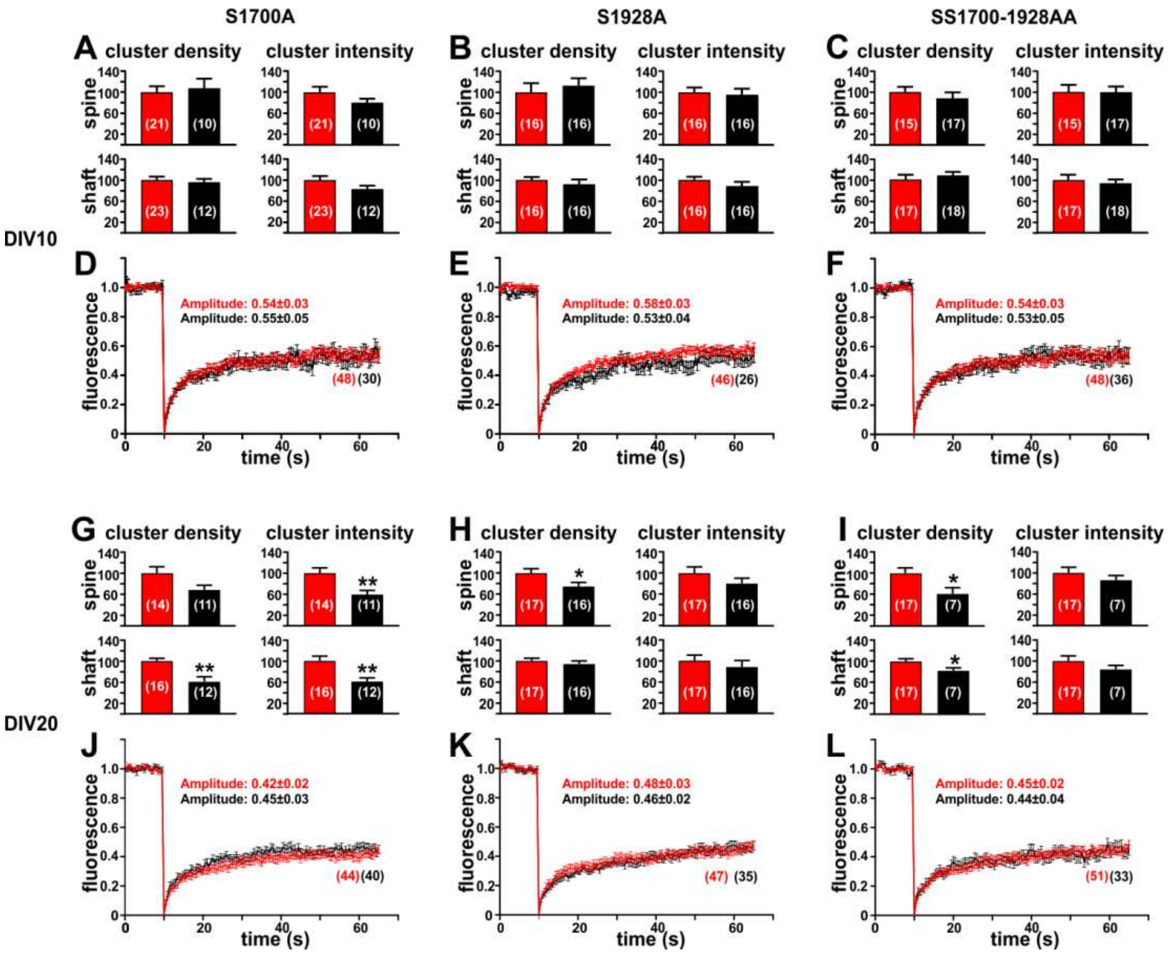

Fig. S4. Quantification of membrane expression and dynamics of phospho-resistant $Ca_V1.2$ channel constructs. (*A-C*, *G-I*) Density and intensity of phospho-resistant HA tagged channels (black) versus control $Ca_V1.2$-HA (red) channel clusters along the dendrites and on the spines. Bar graphs, mean ± SEM; number of analyzed dendritic segments each from a different neuron is in parenthesis. (D-F, J-L) FRAP curves of phospho-resistant eGFP-$Ca_V1.2$-S1700A, eGFP-$Ca_V1.2$-S1928A, and eGFP-$Ca_V1.2$-SS1700-1928AA (all black) are comparable to control eGFP-$Ca_V1.2$ (red). In parenthesis the number of analyzed regions from at least 3 different cultures. Statistics: t-test, *, $0.01<p<0.5$; **, $0.001<p<0.01$.

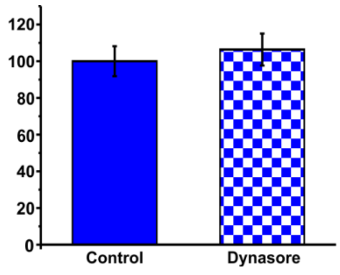

Fig. S5. Density of $Ca_V1.2$-HA-SE clusters along the dendritic shafts upon 30 min of dynasore (80µM) treatment. The number of $Ca_V1.2$-HA channel clusters was previously shown to be up-regulated upon 30 min block of endocytosis (5). In contrast, the $Ca_V1.2$-HA-SE cluster density is insensitive to dynasore induced block of endocytosis indicating that the S1928E mutation likely prevents channel internalization. Experiments were conducted on 3 different cultures. N: Control: 10, Dynasore treated: 11, T-test, p = 0.3.

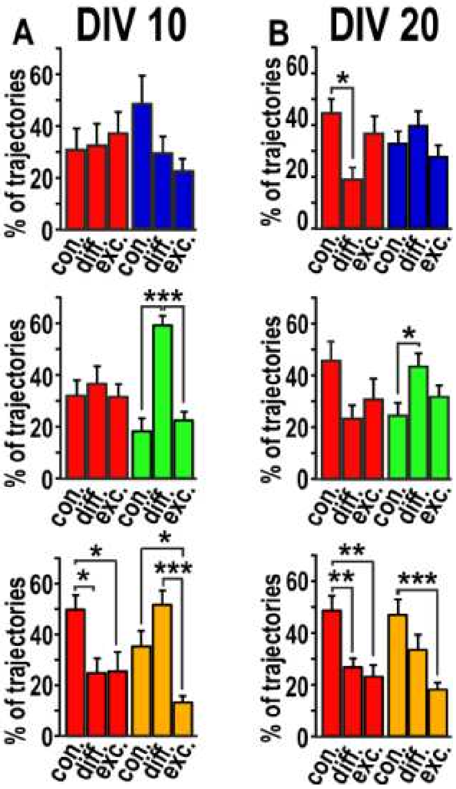

Fig. S6. Modes of trajectories observed for the phospho-mimetic HA tagged $Ca_V1.2$ constructs. Percentage of trajectories displaying confined, diffusive, or mixed modes of lateral mobility in cultured hippocampal neurons at DIV10 (A) and DIV20 (B). Statistics: ANOVA followed by a Tukey post hoc multiple comparison test *, 0.0116<p<0.038; ****, p<0.0001. Bar graphs, mean ± SEM.

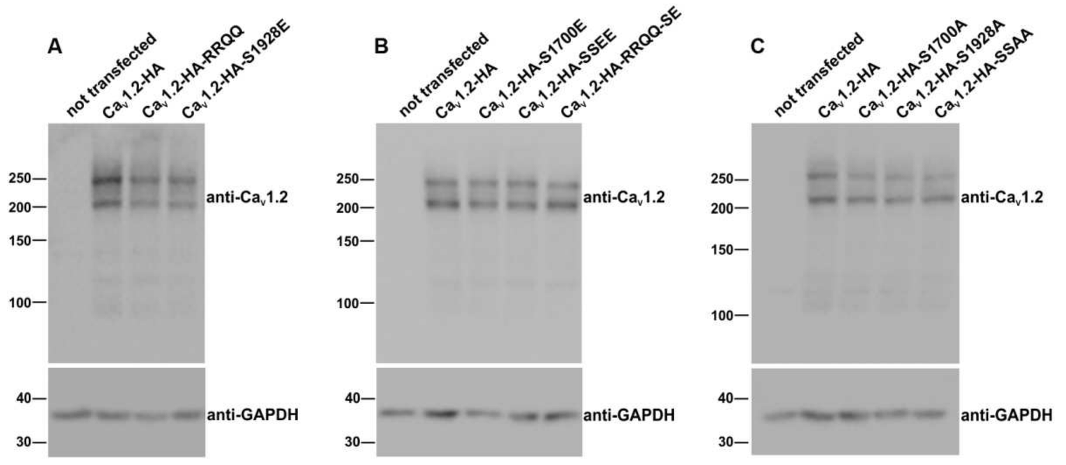

	Ratio cleaved / full length		
	Mean	SD	N
$Ca_V1.2$-HA	0.6	0.4	3
$Ca_V1.2$-HA-S1700A	0.8	0.6	3
$Ca_V1.2$-HA-S1928A	0.8	0.5	3
$Ca_V1.2$-HA-SSAA	0.8	0.7	3

	Ratio cleaved / full length		
	Mean	SD	N
$Ca_V1.2$-HA	0.7	0.4	3
$Ca_V1.2$-HA-RRQQ	0.9	0.5	3
$Ca_V1.2$-HA-S1928E	0.7	0.2	3
$Ca_V1.2$-HA-RRQQ-SE	0.9	0.6	3
$Ca_V1.2$-HA-S1700E	0.8	0.3	3
$Ca_V1.2$-HA-SSEE	0.7	0.4	3

Fig. S7. Representative immunoblots showing the full length and cleaved channels phospho-mimetic (A and B) and phospho-resistant (C) HA-tagged $Ca_V1.2$ constructs used in this study (N=3). HEK293 cells were co-transfected with α2δ1, β_{1a}-eGFP, and the $Ca_V1.2$-HA control and mutants as indicated at the top of each lane. Western blot analysis of the whole cell lysates showed that for all channels two bands corresponding to the full length and cleaved $Ca_V1.2$ were detected by an anti-$Ca_V1.2$ antibody directed to the channel cytoplasmic II-III loop. Thus, the mutations used to generate phospho-mimetic and phospho-resistant channels preserve the proteolytic processing of the C-terminus. The table below shows the ratio between cleaved and full length channel as quantified by densitometric analysis. The double band was detected in each experiment. However, the degree of cleavage was very variable among repetitions. No statistical difference was detected (ANOVA, $p > 0.05$)

Table S1. Analysis of dendritic morphology in neurons expressing $Ca_V1.2$-HA, $Ca_V1.2$-HA-SE, $Ca_V1.2$-HA-RRQQ, and $Ca_V1.2$-HA-RRQQ-SE

	Thickness dendritic shaft (µm)			Number of spines / dendritic µm		
	$Ca_V1.2$-HA	-SE	p	$Ca_V1.2$-HA	-SE	P
d10	1.58±0.3 (19)	1.48±0.4 (26)	n.s.	0.35±0.2 (19)	0.36±0.2 (26)	n.s.
d20	1.42±0.4 (19)	1.3±0.4 (17)	n.s.	0.52±0.2 (19)	0.58±0.2 (17)	n.s.
	$Ca_V1.2$-HA	-RRQQ		$Ca_V1.2$-HA	-RRQQ	
d10	1.49±0.4 (30)	1.42±0.5 (23)	n.s.	0.34±0.2 (30)	0.37±0.2 (23)	n.s.
d20	1.51±0.3 (26)	1.47±0.4 (23)	n.s.	0.58±0.2 (26)	0.62±0.3 (23)	n.s.
	$Ca_V1.2$-HA	-RRQQ-SE		$Ca_V1.2$-HA	-RRQQ-SE	
d10	1.65±0.6 (19)	1.55±0.5 (18)	n.s.	0.14±0.1 (19)	0.2±0.1 (18)	n.s.
d20	1.57±0.6 (22)	1.59±0.7 (20)	n.s.	0.61±0.3 (22)	0.53±0.2(20)	n.s.

Data are presented as Mean±SD; in parenthesis the N number of analyzed dendritic segments which coincide to those analyzed in Fig.2; Number of experiments: at least 3; Statistics: Mann Whitney test between pairs, n.s.: $0.06<p<0.9$
